# Database-Guided Analysis for Immunophenotypic Diagnosis and Follow-Up of Acute Myeloid Leukemia With Recurrent Genetic Abnormalities

**DOI:** 10.3389/fonc.2021.746951

**Published:** 2021-11-05

**Authors:** Carmen-Mariana Aanei, Richard Veyrat-Masson, Cristina Selicean, Mirela Marian, Lauren Rigollet, Adrian Pavel Trifa, Ciprian Tomuleasa, Adrian Serban, Mohamad Cherry, Pascale Flandrin-Gresta, Emmanuelle Tavernier Tardy, Denis Guyotat, Lydia Campos Catafal

**Affiliations:** ^1^ Laboratoire d’Hématologie, Centre Hospitalier Universitaire de Saint-Etienne, Saint-Etienne, France; ^2^ Laboratoire d’Hématologie, Centre Hospitalier Universitaire de Clermont-Ferrand, Clermont-Ferrand, France; ^3^ Department of Hematology, “Iuliu Hațieganu” University of Medicine and Pharmacy, Cluj-Napoca, Romania; ^4^ Laboratory of Hematology, Oncological Institute “Prof. Dr. Ion Chiricuță”, Cluj-Napoca, Romania; ^5^ Department of Medical Genetics, “Iuliu Hațieganu” University of Medicine and Pharmacy, Cluj-Napoca, Romania; ^6^ Department of Genetics, Oncological Institute “Prof. Dr. Ion Chiricuță”, Cluj-Napoca, Romania; ^7^ Department of Clinical Hematology, Oncological Institute “Prof. Dr. Ion Chiricuță”, Cluj-Napoca, Romania; ^8^ Département d’Hématologie Clinique, Institut de Cancérologie Lucien Neuwirth, Saint-Priest-en-Jarez, France

**Keywords:** acute myeloid leukemia with recurrent genetic abnormalities, multicolor flow cytometry, Compass database-guided analysis, different-from-normal (DfN) approach, measurable (minimal) residual disease

## Abstract

Acute myeloid leukemias (AMLs) are hematologic malignancies with varied molecular and immunophenotypic profiles, making them difficult to diagnose and classify. High-dimensional analysis algorithms might increase the utility of multicolor flow cytometry for AML diagnosis and follow-up. The objective of the present study was to assess whether a Compass database-guided analysis can be used to achieve rapid and accurate diagnoses. We conducted this study to determine whether this method could be employed to pilote the genetic and molecular tests and to objectively identify different-from-normal (DfN) patterns to improve measurable residual disease follow-up in AML. Three Compass databases were built using Infinicyt 2.0 software, including normal myeloid-committed hematopoietic precursors (*n* = 20) and AML blasts harboring the most frequent recurrent genetic abnormalities (*n* = 50). The diagnostic accuracy of the Compass database-guided analysis was evaluated in a prospective validation study (125 suspected AML patients). This method excluded AML associated with the following genetic abnormalities: *t*(8;21), *t*(15;17), inv(16), and *KMT2A* translocation, with 92% sensitivity [95% confidence interval (CI): 78.6%–98.3%] and a 98.5% negative predictive value (95% CI: 90.6%–99.8%). Our data showed that the Compass database-guided analysis could identify phenotypic differences between AML groups, representing a useful tool for the identification of DfN patterns.

## 1 Introduction

Acute myeloid leukemia (AML) refers to a heterogeneous group of malignant diseases characterized by the accumulation of aberrant hematopoietic progenitor cells, known as AML blasts, that cannot progress beyond various stages of maturation and are unable to develop into mature blood cells.

According to the current World Health Organization (WHO) criteria (1), an AML diagnosis depends on a combination of clinical findings, morphological evaluations of peripheral blood (PB) and bone marrow (BM) specimens, and cytogenetic (karyotype and fluorescent *in situ* hybridization (FISH)) and molecular analyses [polymerase chain reaction (PCR) and next-generation sequencing (NGS)].

At present, multicolor flow cytometry (MFC) is viewed as a complementary tool that can assist with the AML diagnostic process. MFC is typically used to define the blast cell lineage and can be used to identify phenotypic aberrations, known as leukemia-associated immunophenotypes (LAIPs), such as the presence of aberrant lymphoid markers, maturation asynchrony, or the absence of myeloid markers, which might be useful for assessing measurable residual disease (MRD) during AML treatment follow-up.

The evaluation of cytogenetics and mutational profiles represent reference methods for monitoring MRD in AML, allowing for the assessment of clonal evolution and the stratification of AML into prognostic subgroups to guide treatment approaches (2). Despite the high specificity and sensitivity of PCR-based methods for leukemic cells, their applicability is limited to the approximately 40% of AML patients that harbor one or more traceable molecular abnormalities, according to the European LeukaemiaNet (ELN) MRD Working Party (3). In addition, although complete remission rates have improved in recent years (approaching 80%) due to the application of therapeutic algorithms guided by molecular technologies, greater than 50% of adult patients with AML will undergo disease relapse after initial treatment (2). Therefore, interest exists in the development of MFC applications for disease monitoring in AML, with the potential to perform precise residual disease estimations below the current morphological assessment thresholds for determining complete remission. This method could refine prognostic assessments and direct postremission decision-making processes in AML (2).

However, despite the high applicability of MFC for MRD assessments in AML patients (>90% of all AML cases) compared with molecular MRD assessments (2), multicenter studies have shown a relatively high number of false-positive cases following MFC assessment, resulting in a low specificity of 71%, even when using standardized protocols, which is most likely due to differences in the subjective interpretation of MFC data (4).

To improve AML MRD detection by MFC, the ELN MRD Working Party has recommended combining the different-from-normal (DfN) approach with the LAIP assessment method (3). The major advantage of the DfN approach is that it can be applied even in cases with unknown blast phenotypes at diagnosis and can identify other abnormal immunophenotypic cells, in addition to residual blasts, which is not possible using the LAIP method, which focuses only on the detection of residual blasts carrying the immunophenotypic anomaly identified at diagnosis. Therefore, high-dimensional analysis algorithms may be useful for optimizing the MFC-MRD performance in AML (3).

New tools for MFC data analysis have recently been developed to objectively visualize immunophenotypic differences between abnormal cells from different pathologies. One such tool is Infinicyt Compass, which was developed by the EuroFlow™ (EF) Consortium, and allows for the recognition of complex immunophenotypic patterns through multivariate analyses of flow cytometric data.

The present study aimed to assess whether the Compass database analysis could be used to guide the genetic and molecular testing of AML to achieve a rapid and accurate diagnosis and to perform DfN analyses.

The results of this study showed that the comparison of new cases against reference databases composed of well-classified AML cases represents a user-friendly method that can facilitate the orientation of genetic and molecular biology testing to achieve a rapid, accurate diagnosis. In addition, the Compass database-guided analysis of MFC data can be used as a nonsubjective method for DfN evaluation.

## 2 Materials and Methods

### 2.1 Study Design

**Figure 1:**
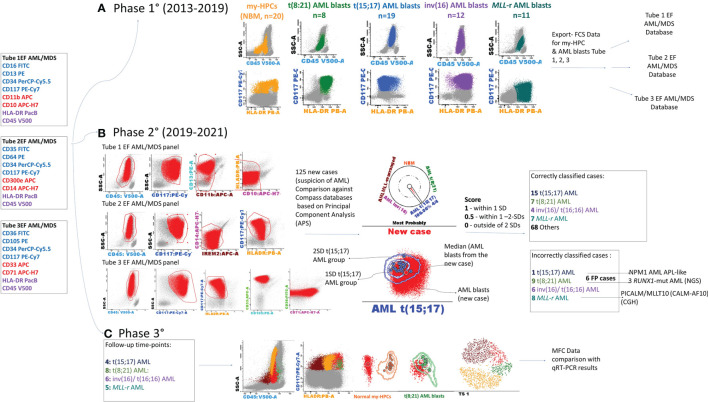
Schematic overview of the study. The first three tubes of the EuroFlow (EF) AML/MDS antibody panel were used for the discrimination of acute myeloid leukemia (AML) blasts and normal myeloid-committed hematopoietic precursors (my-HPCs) in bone marrow aspirates obtained from patients with AML and healthy individuals. **(A)** Phase 1: Construction of the databases. The Compass databases were composed of fcs-exported files corresponding to leukemic blasts from well-classified AML cases, according to the WHO diagnostic recommendations, including *t*(8;21) AML (*n* = 8), *t*(15;17) AML (*n* = 19), inv(16)/*t*(16;16) AML (*n* = 12), AML with *MLL* gene translocations (*MLL*-r AML, *n* = 11), and normal my-HPCs (*n* = 20). **(B)** Phase 2: Samples from 125 patients with suspected AML were compared against the Compass databases based on APS plots. The blast events from each individual AML case were compared against each well-classified AML group and with the normal my-HPC populations using balanced APS plots. The similarity between blast events from the new case and any defined populations was scored based on the position of the median from the new case relative to the 1 and 2 standard deviation (SD) curves for the defined AML groups or normal my-HPC groups included in the database. A total of 101 cases were correctly classified, whereas 24 cases were incorrectly assigned to the wrong AML group or to the other group. The incorrectly assigned *t*(15;17) AML case was an *NPM1* ^+^ AML with an acute promyelocytic leukemia (APL)-like phenotype; the three false-positive *t*(8;21) AML cases were *RUNX1*-mutant AML cases, and for the false-positive *MLL*-r AML case, the array-comparative genomic hybridization (CGH) analysis identified a *PICALM/MLLT10* (*CALM-AF10*) fusion gene. **(C)** Phase 3: Evaluation of the Compass database-guided DfN analysis. Immunophenotypic data acquired at different MRD follow-up time points from four AML patients with different genetic abnormalities who were in cytological remission were used, including one *MLL*-r AML case, one *t*(8;21) AML case, one *t*(15;17) AML case, and one inv(16) AML case. Compass database-guided analysis was compared against quantitative reverse transcription–polymerase chain reaction (qRT-PCR).

The present study was conducted in three phases: construction of Compass databases; databases-guided analysis of new AML cases at initial diagnosis; and evaluation of database-guided DfN analysis (**Figure 1**).

### 2.2 Construction of the Databases

**Figure 2:**
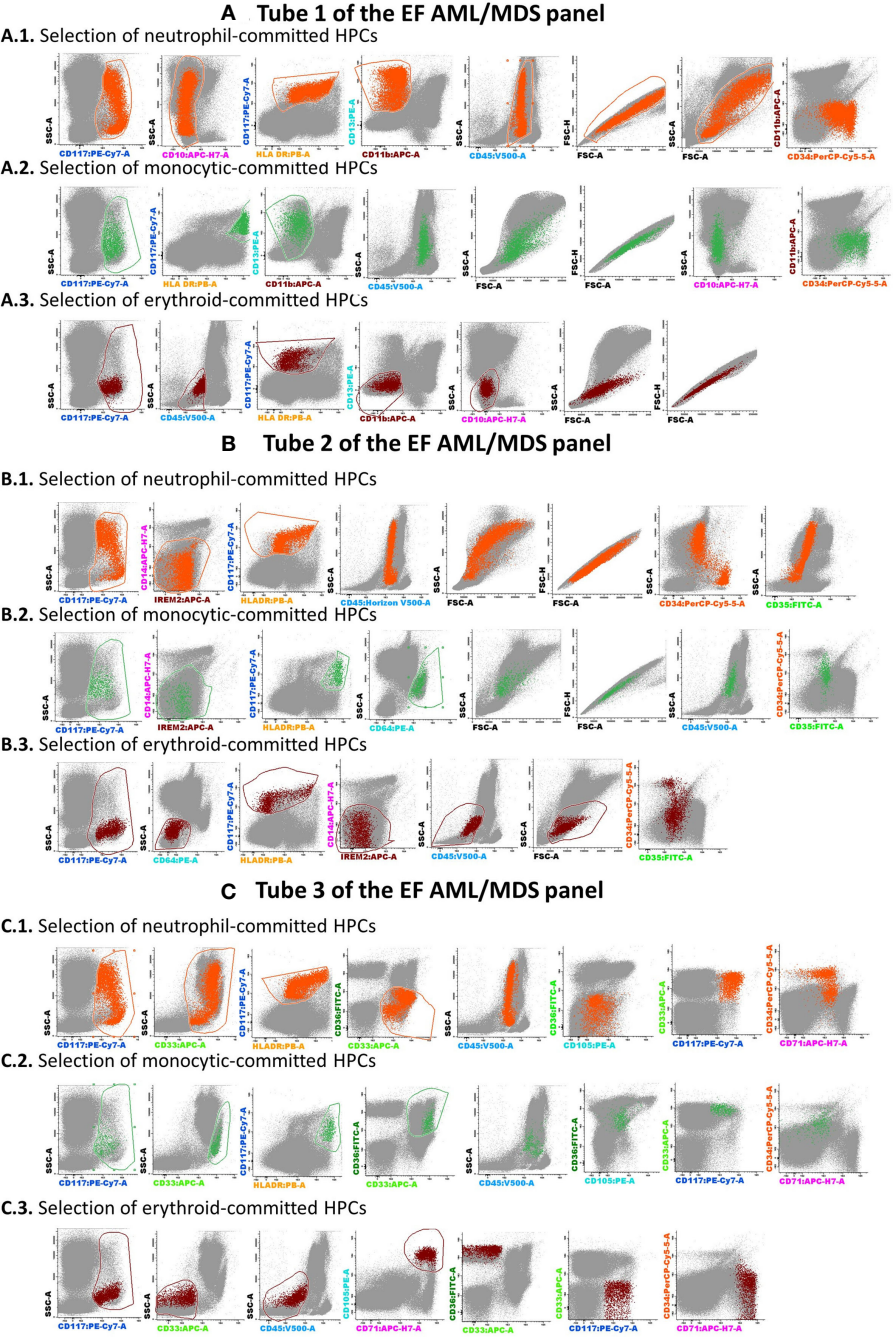
The analysis strategy for the identification of normal myeloid-committed HPCs using Tubes 1–3 of the EF AML/MDS panel. The discrimination of normal my-HPCs was performed by Infinicyt 2.0 software, based primarily on the backbone markers CD117, human leukocyte antigen-DR (HLA-DR), and CD45. Several exclusion gates were used to avoid the inclusion of undesirable events that may fall into the blast gate, such as CD11b^+^ hypogranular neutrophils and basophils (Tube 1), CD14^+low^ granulocytes and CD14^+^ CD300e (IREM-2)^+^ monocytes (Tube 2), and CD10^+^ hematogones (Tube 1). Bivariate dot plot histograms illustrating the HPCs committed toward a neutrophil lineage (CD45^+low/int^CD117^+^CD34^+/−^HLA-DR^+int^CD13^+^CD14^−^ IREM2^−^CD33^+^CD36^−^; orange dots), a monocyte lineage (CD45^+low/int^CD117^+^CD34^+/−^HLA-DR^+high^CD64^+^CD14^−^CD300e/IREM-2^−^CD13^+^CD33^+^; green dots), or an erythroid lineage (CD45^+low/−^CD117^+^CD34^+/−^HLA-DR^+low/int^CD36^+^CD105^+^CD33^−^CD35^+low^; dark-red dots). The other bone marrow cells are displayed in gray. **(A)** tube 1 EF AML/MDS panel; **(B)** tube 2 EF AML/MDS panel, **(C)** tube 3 EF AML/MDS panel.

The Compass databases are composed of fcs-exported data files featuring characteristics of leukemic blasts from well-classified AML cases, according to WHO recommendations (1), including *t*(8;21) AML (*n* = 8), *t*(15;17) AML (*n* = 19), inv(16)/*t*(16;16) AML (*n* = 12), and AML with *MLL* gene translocations (*MLL*-r AML, *n* = 11); all samples were diagnosed by the Hematology Laboratory from the University Hospital of Saint-Etienne between 2013 and 2019. Normal myeloid hematopoietic precursor cells (my-HPCs) committed toward a neutrophil lineage (CD45^+low/int^ CD117^+^ CD34^+/−^ HLA-DR^+int^ CD13^+^ CD14^−^ IREM2^−^ CD33^+^ CD36^−^), a monocyte lineage (CD45^+low/int^ CD117^+^ CD34^+/−^ HLA-DR^+hi^ CD64^+^ CD14^−^ CD300e[IREM-2]^−^ CD13^+^ CD33^+^), and an erythroid lineage (CD45^−/+low^ CD117^+^ CD34^+/−^ HLA-DR^+low/int^ CD36^+^ CD105^+^ CD33^−^ CD35^+low^) were also included (**Figure 2**). The immunophenotypic characterization of my-HPCs using the EF AML/MDS panel was performed in the present study using normal BM samples obtained from healthy individuals with normal blood counts (eleven healthy BM donors (HDs) and nine patients undergoing sternotomy for cardiac surgery (CS)) from the University Hospitals of Saint-Etienne and Clermont-Ferrand, France. The strategy used for the selection of normal my-HPCs was based on recently published data (5, 6). Pregating for intact singlets, followed by the discrimination of normal my-HPCs, was performed using the Infinicyt 2.0 software based primarily on the backbone markers CD117, HLA-DR, and CD45, in addition to several lineage-specific markers, such as CD13 for neutrophil-committed HPCs; CD64 and CD33 for monocyte-committed HPCs; and CD105 and CD36 for erythroid-committed HPCs (**Figure 2**). Several exclusion gates were used to avoid the inclusion of undesirable events that may fall into the CD45^+low^ CD117^+^ HLA-DR^+^ blast gate, such as CD11b^+^ hypogranular neutrophils and basophils (tube 1), CD14^+low^ granulocytes and CD14^+^ IREM-2^+^ monocytes (tube 2), and CD10^+^ hematogones (tube 1) (**Figure 2**). Three databases corresponding to the first three tubes of the EF AML/MDS panel which are dedicated to the immunophenotypic analysis of the principals myeloid lineages were built from merged fcs files containing AML blasts from the four AML groups and the normal my-HPCs: tube 1 of the EF AML/MDS panel (neutrophil lineage), tube 2 of the EF AML/MDS panel (monocytic lineage), and tube 3 of the EF AML/MDS panel (erythroid cell lineage). The fcs files used to build the databases are available at FlowRepository (ID: FR-FCM-Z3JL).

Each group of cases was subsequently plotted in a balanced automatic population separator (APS), a principal component analysis (PCA) plot for comparisons with other groups included in the databases.

### 2.3 Database-Guided Analysis of New AML Cases at Initial Diagnosis

The BM samples used in this prospective study were obtained between January 2019 and June 2021 from 125 consecutive patients who were hospitalized with suspected AML at the Institut de Cancérologie Lucien Neuwirth, Saint-Priest-en-Jarez, the Estaing University Hospital of Clermont-Ferrand, France, and the Oncological Institute Prof. Dr. Ion Chiricuță, Cluj-Napoca, Romania.

Written informed consent was obtained from each patient and healthy donor (HD), as approved by the institutional procedures of the independent ethics committee and the Comité de Protection des Personnes - Ile de France (NCT03233074/17.07.2017).

All participants’ characteristics are summarized in **Table 1** and detailed in **Table S1**.

**Table 1:** Characteristics of patients used for evaluation of the database-guided analysis.

	Parameter	Validation cohort	Database^a^
WHO diagnosis (*n*)	APL with PML-RARA	15	19
	AML with *t*(8;21)(q22;q22.1);RUNX1-RUNX1T1	7	8
	AML with inv(16)(p13.1q22) or *t*(16;16)(p13.1;q22);CBFB-MYH11	6	12
	KMT2A(MLL)-rearranged AML	7	11
	AML with mutated NPM1	16	
	*Provisional entity: AML with mutated RUNX1*	6	
	AML with biallelic mutations of CEBPA	1	
	AML-MRC	41	
	AML NOS	24	
	Blastic plasmacytoid dendritic cell neoplasm	1	
	Aggressive NK leukemia/lymphoma	1	
Gender	F	58	23
	M	67	27
Age (years)	Median	66	51
	Range	13—94	1–83
WBC (10^9^/L)	Median	33.5	37.6
	Range	0.6–537.8	0.8–308.7

AML, acute myeloid leukemia; AML-MRC, AML with myelodysplasia-related changes; APL, acute promyelocytic leukemia; AML NOS, AML, not otherwise specified. ^a^Additional details can be found in **Table S1**.

The phenotypes of leukemic blasts obtained from new patients were compared against the phenotypes of AML blasts from four different cytogenetic groups and the most similar my-HPCs using the three databases.

**Figure 3:**
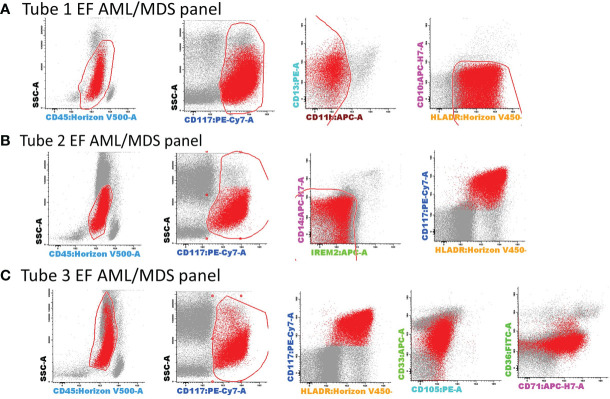
Gating strategy for the selection of AML blasts using tubes 1–3 of the EF AML/MDS panel. AML blasts were selected based on the expression of backbone markers, CD117, human leukocyte antigen-DR (HLA-DR), and CD45^+low^. Several exclusion gates were used to avoid the inclusion of undesirable events that may fall into the blast gate, such as CD11b^+^ hypogranular neutrophils and basophils (tube 1), CD14^+low^ granulocytes and CD14^+^CD300e (IREM-2)^+^ monocytes (tube 2), and CD10^+^ hematogones (tube 1). Bivariate dot plots illustrating a representative example of AML blast identification using the antibody combinations from tubes 1–3 of the EF AML/MDS panel. AML blasts (red dots), other singlet events (gray dots). **(A)** tube 1 EF AML/MDS panel; **(B)** tube 2 EF AML/MDS panel, **(C)** tube 3 EF AML/MDS panel.

AML blasts were selected according to CD45^+low^ expression, CD117, HLA-DR positivity, and side scatter (SSC) characteristics, as previously described (7). Several exclusion gates were applied to avoid the inclusion of undesirable events, such as CD11b+ hypogranular neutrophils and basophils (tube 1), CD14^+low^ granulocytes and CD14^+^ IREM-2^+^ monocytes (tube 2), and CD10^+^ hematogones (tube 1) (**Figure 3**).

The AML blast events identified in each individual AML case were compared with each well-classified AML group and normal my-HPC populations using balanced APS plots.

**Figure 4:**
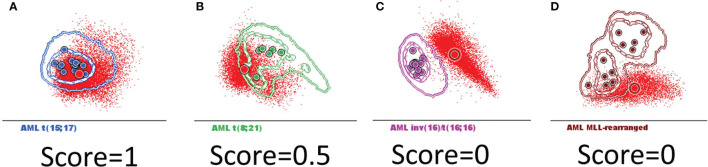
The scoring system used to determine whether AML blasts from a new case can be classified in the defined AML groups harboring recurrent genetic abnormalities that were included in the Compass databases. Acute myeloid leukemia (AML) blasts from a new case (red dots and circles) were compared with the AML groups included in the databases: *t*(15;17) AML (blue), *t*(8;21) AML (green), inv(16)/*t*(16;16) AML (violet), and *MLL*-r AML (dark red). The circles represent the median values of individual cases. The dotted line represents the 1 standard deviation (SD) curve, and the solid line represents the 2 SD curve for the AML group. The similarity between the two populations was scored based on the position of the median for the new case blast events relative to the 1 and 2 SD curves for the AML groups that were included in the database: **(A)** falling within 1 SD was scored as 1 point; **(B)** falling within 1–2 SDs was scored as 0.5 points; and **(C, D)** falling outside of 2 SDs was scored as 0 points.

The similarity between any two populations was scored based on the position of the median for the new AML blast events from each case relative to the 1 and 2 standard deviation (SD) curves for the AML groups and normal my-HPCs included in the database: falling within 1 SD was scored as 1 point (**Figure 4A**); falling within 1–2 SDs was scored as 0.5 point (**Figure 4B**); and falling outside of 2 SDs was scored as 0 points (**Figures 4C, D**).

After comparison with the AML phenotypes in the databases, the new AML cases were classified as follows:

*Typical, clearly belonging to an AML group* was used to described AML blast events that fell within 1 or within 1–2- SDs of a single AML group and outside of 2 SDs for all other groups based on the results of at least two of the three tubes from the EF AML/MDS panel (**Figure S1A**).

*Atypical, oriented toward an AML group* was used to describe AML blast events that fell within 1 SD or within 1–2 SDs for two or more AML groups; the case was assigned to the group with the highest score obtained after combining the scores obtained for each of the three tubes of the EF AML/MDS panel (**Figure S1B**).

*Other* was used to describe AML blast events that fell outside of 2 SDs for all AML groups or within 1–2 SDs of any AML group based on the results of only one of the three tubes from the EF AML/MDS panel (**Figure S1C**).

The Compass database-guided results were compared with the final diagnoses for all suspected AML cases, which were established using a combination of morphological aspects, karyotypes, FISH analysis, and molecular biology data, according to WHO recommendations (1).

The results of comparisons between database-guided classifications and the final clinical diagnoses were classified as follows: *true positives*, when the AML group indicated by the Compass database-guided approach coincided with the final diagnosis; *false positives*, when the AML group indicated by the Compass database-guided approach was not confirmed by other diagnostic tests; *false negative*, when a final diagnosis was made in favor of an AML group featuring a genetic abnormality that was not identified by the Compass database-guided approach; and **true negative**, when the Compass database-guided approach did not identify an immunophenotype corresponding to any AML groups with recurrent genetic abnormalities, and no FISH or PCR tests detected any of the genetic abnormalities assessed in the study.

The false-positive and false-negative AML cases were thereafter re-evaluated using a neighborhood automatic population separator (NAPS) PCA-based plot.

### 2.4 Database-Guided DfN

We sought to evaluate whether the database-guided analysis could be used to identify DfN immunophenotypes during AML follow-up. We evaluated immunophenotypic data acquired at different MRD follow-up moments from four AML patients with different genetic abnormalities, including five time points for one with *MLL*-r AML, eight time points for one with *t*(8;21) AML, four time points for one with *t*(15;17) AML, and six time points for one with inv(16) AML. Nondebris, singlet, CD117^+^ HLA-DR^+^ my-HPCs were selected from each of the fcs files analysed for each patient, as described above (**Figure 2**). Events were evaluated for inclusion into one of the following groups, based on comparison with the developed Compass databases: normal my-HPCs, AML *t*(8;21), AML inv(16), AML *t*(15;17), and *MLL*-r AML (**Figures S2**–**S5**).

Events that fell outside of 2 SDs for normal my-HPCs but within 2 SDs of corresponding AML group were considerate DfN and classified into the AML blast group.

MFC data were compared with the results of quantitative reverse transcription-PCR (qRT-PCR).

### 2.5 Immunophenotyping

All samples were stained using the first three tubes of the EF AML/MDS antibody panel (7). Sample preparation and acquisition were performed according to the EF standard operating procedure and using the recommended EF instrument settings (8).

Appropriate instrument performance was confirmed by performing FranceFlow and EuroFlow quality assessments (9, 10).

At least 100,000 BM cells/tube were acquired using a three-laser, eight-color BD FACSCanto-II™ flow cytometer (BD Bioscience, San José, CA, USA) at each study site. Acquired cells were then analyzed using Infinicyt V2.0 (Cytognos, Salamanca, Spain).

### 2.6 Morphologic Examination

May-Grünwald-Giemsa-stained BM aspirate smears from each AML patient were examined under a light microscope by experienced pathologists and the diagnosis was established conforming WHO 2016 guidelines (1).

### 2.7 Cytogenetics Analysis

BM samples were obtained from all AML patients for cytogenetic (CG) analysis at the time of diagnosis. Karyotypes were analyzed after 24 h of unstimulated culture using standard procedures. The chromosomes were stained by R- and G-banding. At least 20 metaphase events were analyzed. The results were interpreted and reported according to the International System for Human Cytogenetic Nomenclature (ISCN, 2013 and 2016) (11).

FISH was performed for promyelocytic leukemia/retinoic acid receptor α (*PML-RARA*; dual-color, dual-fusion probe, Abbott, Des Plaines, IL, USA), Runt-related transcription factor/RUNX1 partner transcriptional corepressor 1 (*RUNX1/RUNX1T1*; dual-color, dual-fusion probe, Metasystems Probes, Altlussheim, Germany), core-binding factor subunit beta (*CBFB*; dual-color, break-apart probe, Metasystems Probes, Altlussheim, Germany), and lysine methyltransferase 2A (*KMT2A*; dual-color, break-apart probe, Metasystems Probes, Altlussheim, Germany). Detection was performed on freshly harvested BM cells (metaphase and interphase). Twenty metaphase events and 200 nuclei were observed for each case.

### 2.8 Quantitative Reverse Transcription-Polymerase Chain Reaction

Multiparametric RT-PCR was performed to detect recurrent fusion transcripts in all newly diagnosed AMLs. In cases positive for any fusion products, qRT-PCR was performed to monitor treatment response. The panel tests included *PML/RARA*, *RUNX1-RUNX1T1 (AML1-ETO)*, *CBFB-MYH11* variant A, and *CBFB-MYH11* variant D. In brief, extracted RNA was analyzed by RT-PCR for the *PML/RARA*, *RUNX1-RUNX1T1 (AML1-ETO)*, and *CBFB-MYH11* fusion transcripts on an ABI HT platform (Applied Biosystems, Villebon Sur Yvette, France) and on an ABI 3500 DNA analyzer (Applied Biosystems, Thermo Fisher, Waltham, MA, USA). Quantitative values are expressed as a ratio of the fusion transcript level to the *ABL1* transcript level (%), and the sensitivity was determined to be 0.001%.

Wilms’ tumor gene (*WT1*) expression levels were quantified in PB samples using qRT-PCR and normalized against total *ABL1* gene expression levels to monitor MRD for *KMT2A*(*MLL*)-rearranged AML cases.

### 2.9 Next-Generation Sequencing

Genomic DNA was tested for most AML cases at diagnosis by NGS using a custom-designed myeloid panel. The panel assessed 51 commonly mutated genes associated with myeloid malignancies: *ATM*, *ASXL1*, *BCOR*, *BCORL1*, *CALR*, *CBL*, *CEBPa*, *CSF3R*, *DDX41*, *DNMT3A*, *EPOR*, *ETNK1*, *ETV6*, *EZH2*, *FLT3*, *GATA1*, *GATA2*, *GNAS*, *HRAS*, *JAK2*, *IDH1*, *IDH2*, *KRAS*, *KDM6A*, *KIT*, *MPL*, *NF1*, *NFE2*, *NPM1*, *NRAS*, *PHF6*, *PPM1D*, *PTPN11*, *RAD21*, *RUNX1*, *SF3B1*, *SETBP1*, *SH2B3*, *SMC1A*, *SMC3*, *SRSF2*, *STAG1*, *STAG2*, *STATB5*, *TET2*, *THPO*, *TP53*, *U2AF1*, *U2AF2*, *WT1*, and *ZRSR2*.

Briefly, libraries were obtained by hybrid capture-based target enrichment (SureSelectXT Low Input, Agilent, Santa Clara, CA, USA), paired-end sequencing was performed on an Illumina MiSeq System (Chip V2-300; Illumina, San Diego, CA, USA), and sequence analysis was realized on an outsourced bioinformatics solution SeqOne (Hg19 alignment).

### 2.10 Statistical Analysis

A receiver operator characteristic (ROC) curve was generated to determine the ability of the Compass database-guided analysis to correctly classify new AML cases based on the established scoring system for this study.

The ROC curve, area under the curve (AUC), sensitivity, specificity, positive and negative predictive values, and likelihood ratios were estimated using MedCalc Software Ltd. Version 19.8 (Ostend, Belgium).

## 3 Results

### 3.1 Prospective Validation Study

The resulting AML databases and the database-guided analysis tool were validated on 125 consecutive AML-suspected cases. The results were compared with the final diagnosis according to WHO 2016 guidelines that were established by each center. Overall, the distribution of the 125 cases across the various AML groups was as follows: 15 (12%) t(15;17) AML; 7 (5.6%) t(8;21) AML; 6 (4.8%) inv(16)/t(16;16) AML; 8 (6.4%) KMT2A(MLL) AML; 88 (70.4%) other AML; and 1 (0.8%) non-AML. The distribution across the database-guided result categories (typical, atypical, or other), based on the scoring algorithm described in the *Materials and Methods* section, is shown in **Table 2**.

**Table 2:** Results of the database-guided analysis per AML category.

Compass result	AML category^a^				
	*t*(15;17) AML	*t*(8;21) AML	inv(16) AML	KMT2A(MLL) AML	Other
Typical	8 (6.4%)	6 (4.8%)	4 (3.2%)	3 (2.4%)	62 (49.6%)
Atypical	7^b^ (5.6%)	1^b^ (0.8%)	0^b^ (0%)	4^b^ (3.2%)	5^c^ (4%)
Other	0 (0%)	0 (0%)	2 (1.6%)	1 (0.8%)	21^d^ (16.8%)

AML, acute myeloid leukemia; t(15;17) AML, APL with PML-RARA; t(8;21) AML, AML with t(8;21)(q22;q22.1);RUNX1-RUNX1T1; inv(16) AML, AML with inv(16)(p13.1q22) or t(16;16)(p13.1;q22);CBFB-MYH11; MLL AML, KMT2A(MLL)-rearranged AML.

a Number of cases and percentages within each AML category.

b Cases were the median of AML blast events fall within the 1 SD or 1–2 SDs of two or more AML groups; the assignment in a group being realized on the highest score obtained by summarizing the individual scores obtained in each of the three tubes.

c Uncertain cases were the median of AML blast events fall within the 1–2 SD of two or more AML groups and the final score for each AML group was less than 2.

d False-positive cases in the “Other” group.

**Figure 5:**
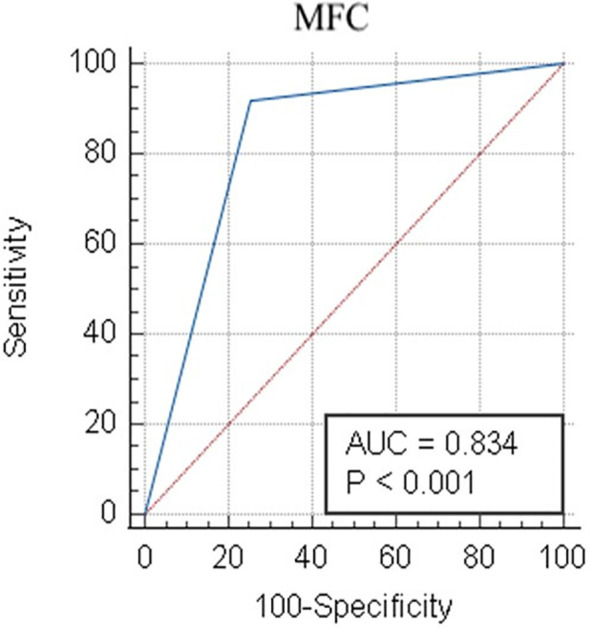
Receiver operator characteristic evaluation of the performance of the Compass database-guided analysis for the correct classification of AML cases. Receiver operating characteristic (ROC) curve (blue line) comparing the results of the Compass database-guided analysis with those provided by FISH or PCR tests. The red diagonal line represents a random classifier. AML, acute myeloid leukemia; MFC, multicolor flow cytometry; AUC, area under the curve.

When combining the Compass database-guided results obtained from the first three tubes of the EF AML/MDS panel, a true positive result was obtained in 33 (26.4%) cases, a true negative result was obtained in 67 (53.6%) cases, a false-positive result as obtained in 24 (19.2%) cases, and a false-negative result was obtained in one (0.8%) case. The Compass database-guided diagnosis allowed for correct classification with an AUC of 0.83 (95% confidence interval (CI): 0.75–0.89; *p* < 0.001; **Figure 5**). Overall, this method was able to exclude AML associated with *t*(8;21), *t*(15;17), inv(16)/*t*(16;16), and *KMT2A*(*MLL*) translocation with 92% sensitivity (95% CI: 78.6%–98.3%), a 98.5% negative predictive value (95% CI: 90.6%–99.8%), and a likelihood ratio for a negative test of 0.04 (95% CI: 0.01–0.28). The negative predictive value was 100% for t(15;17) and t(8;21) AMLs; and 99.1% (95% CI: 95.2%–99.9%) for inv(16)/t(16;16) and *KMT2A*(*MLL*) AMLs (95% CI: 94.6%–99.9%). Globally, a reduced positive predictive value (PPV) of approximately 58% (95% CI: 49%–66%) was observed [PPV*_t_*_(15;17)AML_: 94% (95% CI: 68%–99%); PPV*_KMT2A(MLL)_*_AML_: 47% (95% CI: 30%–64%); PPV*_t_*_(8;21)AML_: 44% (95% CI: 29%–59%), and PPV_(inv16)/_ *_t_*_(16;16)AML_: 40% (95% CI: 21%–62%)].

A relatively increased number of false-positive cases were observed for the following AML groups: *t*(8;21) AML (9/118 negative cases), inv(16)/t(16;16) AML (6/119 negative cases), and *KMT2A*(*MLL*) AML (8/117 negative cases).

### 3.2 Detailed Evaluation of Discordant Cases

#### 3.2.1 Re-Evaluation of the False-Positive *t*(8;21) AML Cases

**Figure 6:**
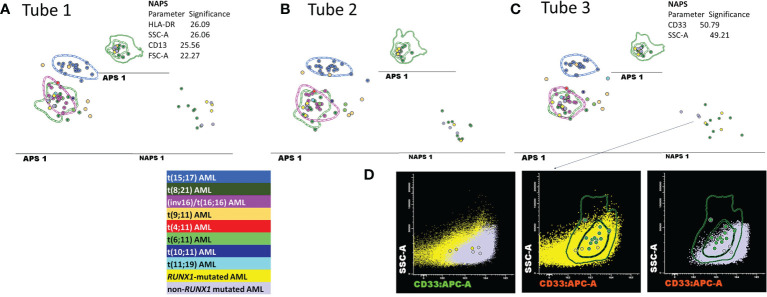
Detailed evaluation of the false-positive *t*(8;21) AML cases. **(A–C)** APS plots showing the perfect overlap between the medians for the blast events from the false-positive *t*(8;21) AML cases (bright yellow and mauve circles) with those for the *t*(8;21) AML group (green circles, the dotted line represents the 1 standard deviation (SD) curve for the group) and for the inv(16)/*t*(16;16) AML group (violet circles, the dotted line represents the 1 SD curve of the group) using the first three tubes of the EF AML/MDS panel. In the tube 3 of EF AML/MDS panel, the neighborhood automatic population separator (NAPS) diagrams allow for the division of false-positive *t*(8;21) AML cases into two groups, according to the presence (bright yellow circles) or absence (mauve circles) of RUNX1 mutation according to NGS analysis. The tables show the contributions of each parameter to the separation of the false-positive *t*(8;21) AML blasts from the *t*(8;21) AML group in the NAPS diagrams, reflected as percentages. **(D)** Bivariate dot plots illustrating the differences in the CD33 and SSC parameters between *RUNX1*-mutated cases (yellow dots and circles representing the median CD33 expression for an individual case) and *RUNX1* nonmutated cases (mauve dots and circles representing the median CD33 expression for an individual case) compared with *t*(8;21) AML cases (green circles representing the median CD33 expression for an individual case; the dotted line represents the 1 SD curve, and the solid line represents the 2 SD curve for the AML group).

Despite the perfect correspondence between the phenotypes of six discordant *t*(8;21) AML cases with the typical *t*(8;21) AML cases on the APS plots (the median of the blast events from these cases fell within 1 SD for the *t*(8;21) AML group), the NAPS diagrams (12) enabled the identification of several differences (**Figure 6**). NAPS showed clear distinctions between *t*(8;21) AML cases and these false-positive *t*(8;21) AML cases (except AML case 35) for markers from tube 1 of the EF AML/MDS panel, based on different patterns for HLA-DR (26%), and CD13 (25%) and differences in the SSC (26%) and forward scatter (FSC, 22%) parameters (**Figure 6A**). In addition, for markers in tube 3 of the EF AML/MDS panel, NAPS identified two distinct groups among the false-positive *t*(8; 21) AML cases, based on differences in CD33 and SSC (**Figure 6C**). Remarkably, when analyzing the NGS data for these cases, the cases closer to the *t*(8;21) AML group were found to harbor a somatic mutations clustering within the Runt domain (*RUNX1*-mutated AML cases: 36, 53, and 66; *RUNX1* nonmutated AML cases: 11, 35, and 74; **Table S1**). Of note, CD33 expression was lower in *RUNX1*-mutated cases, similar to that observed for *t*(8;21) AML blasts.

#### 3.2.2 Re-Evaluation of the False-Positive inv(16)/*t*(16;16) AML Cases

**Figure 7:**
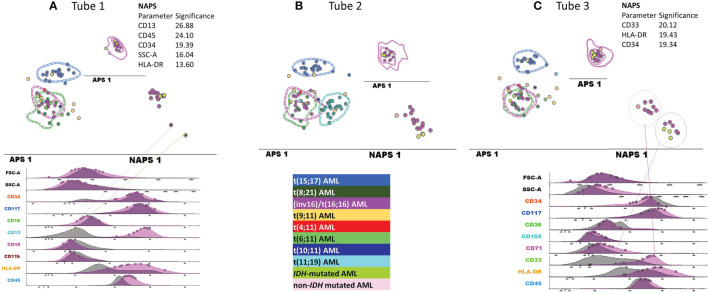
Detailed evaluation of the false-positive inv(16)/t(16;16) AML cases. APS plots showing the overlap between the medians for the blast events from the false-positive inv(16)/t(16;16) AML cases (green-yellow, IDH mutated and pale rose circles, non-IDH mutated) with those for the inv(16)/t(16;16) AML group (violet circles, the dotted line represents the 1 standard deviation (SD) curve for the group), the t(8;21) AML group (green circles, the dotted line represents the 1 SD curve for the group) using the first three tubes of the EF AML/MDS panel **(A)** tube 1 EF AML/MDS panel; **(B)** tube 2 EF AML/MDS panel, **(C)** tube 3 EF AML/MDS panel.

When examining the false-positive inv(16)/t(16;16) AML cases, the NAPS diagram (**Figures 7A–C**) allowed for the identification of AML cases lacking the expression of CD13 or CD33 expression (**Figure 7A, C**); however, this difference in expression was not associated with any specific mutation patterns conforming with the NGS tests.

#### 3.2.3 Re-Evaluation of the False-Positive *MLL*-r AML Cases

**Figure 8:**
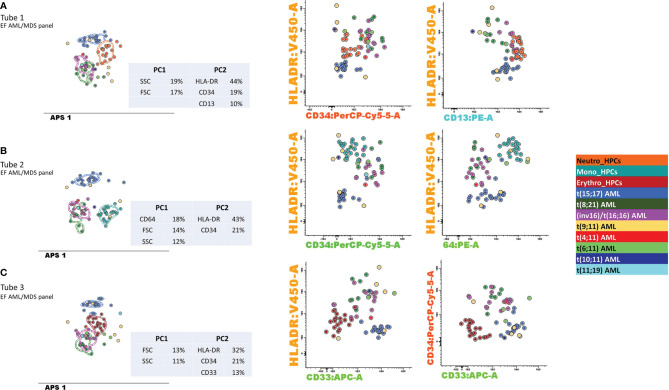
Contribution of markers to the separation of blasts from different AML groups with recurrent genetic abnormalities and myeloid-committed normal HPCs. Samples were stained with antibodies from tubes 1–3 of the EuroFlow (EF) acute myeloid leukemia (AML)/myelodysplastic syndrome (MDS) panel. Bivariate dot plots and principal component analysis (APS) diagrams for 20 neutrophil-committed HPCs (orange), 20 monocyte-committed HPCs (turquoise), 20 erythroidcommitted HPCs (dark red), 19 t(15;17) AML cases (blue), 8 t(8;21) AML cases (dark green), 13 inv(16)/t(16;16) AML cases (violet), 5 t(19;11) AML cases (yellow), 1 t(4;11) AML cases (red), 3 t(6;11) AML cases (light green), 1 t(10;11) AML cases (dark blue), and 2 t(11;19) AML cases (light blue). showed good separation between neutrophil-committed HPCs and AML blasts using tube 1 of the EF AML/MDS panel, based on HLA-DR, CD34, and CD13 expression; between monocyte-committed HPCs and AML blasts using tube 2 of the EF AML/MDS panel, based on CD64, HLA-DR, and CD34 expression and between erythroid-committed HPCs and AML blasts using tube 3 of the EF AML/MDS panel, based on HLA-DR, CD34, and CD33 expression. The circles represent the median values of the blast events for an individual case. The dotted line represents the 1 standard deviation (SD) curve for the group, and the solid line represents the 2 SD curve. The tables show the contributions of each parameter to the first (PC1, *x*-axis) or second (PC2, *y*-axis) principal components, reflected as percentages. **(A)** tube 1 EF AML/MDS panel; **(B)** tube 2 EF AML/MDS panel, **(C)** tube 3 EF AML/MDS panel.

The wide variety of genetic abnormalities identified in the *MLL*-r group was reflected by large phenotypic variability (**Figure 8**), resulting in limited specificity and PPV values and an increased rate of false-positive results.

An interesting example from the *MLL*-r false-positive group is AML case 37 (**Figure S6**). A young adult woman patient presenting with multiple venous thromboses was hospitalized under emergency circumstances at the University Hospital of Saint-Etienne. A hemoglobin level of 6.4 g/dl was detected during a complete blood count test, and the PB smear examination revealed the presence of 64% blasts. Hemostasis tests revealed a D-dimer level >20,000 ng/ml, hypofibrinogenemia, and increased fibrin monomers >150 µg/ml. These findings resulted in suspicion of acute promyelocytic leukemia (APL), and complementary testing of the BM aspirate was performed with urgency. The morphological examination revealed the presence of undifferentiated blasts, and the immunophenotypic evaluation ruled out the *t*(15;17) AML phenotype. The Compass database-guided analysis guided the diagnosis toward the *MLL*-r AML group. Genetic and molecular tests confirmed the absence of *t*(15;17)(q24;q21) or *PML/RARA* rearrangement; however, the karyotype and FISH analyses did not reveal the presence of an *MLL* rearrangement, and an array-comparative genomic hybridization (CGH) analysis was performed. A *PICALM/MLLT10 (CALM-AF10*) fusion gene was identified.

#### 3.2.4 Re-Evaluation of the False-Positive *t*(15;17) AML Case

An 80-year-old female patient (AML case 50) was transferred from a peripheral hospital to the University Hospital of Saint-Etienne with suspicion of APL. The hemostasis screening tests revealed a decrease in the prothrombin time and hypofibrinogenemia. Complementary tests were performed with urgency on PB and BM aspirate samples. Despite a preliminary orientation toward *t*(15;17) AML by the Compass database-guided analysis of the MFC results (**Figure S7**), cytogenetics analysis revealed a 46,XX,del(20)(q11q13)[20] karyotype, with no evidence of *PML-RARA* mutations by FISH or RT-PCR. NGS revealed pathogenic variants in nucleophosmin 1 (*NPM1*; type A) and FMS-like tyrosine kinase 3-internal tandem duplication (*FLT3-ITD*). An APL‐like immunophenotype was previously described for AML with mutated *NPM1*, associated with significantly longer relapse‐free survival compared with patients lacking this phenotype (13) and a good response to all-trans retinoic acid (ATRA) therapy (14).

### 3.3 Contribution of Markers to the DfN Identification

The discriminatory power to distinguish between the different AML groups and normal my-HPCs included in the databases was assessed for the performance of DfN analysis using the Compass database-guided analysis. Tested antibody combinations were evaluated using balanced APS plots, using all eight markers and the FSC and SSC parameters for each tube of the EF AML/MDS panel (**Figure 8**).

Antibody combinations from the three tubes only provided a clear distinction between the blasts from different AML groups was for the *t*(15;17) AML group. Interestingly, a good separation was also observed between *t*(15;17) AML blasts and normal neutrophil-committed CD13^+^ HPCs (tube 1 of the EF AML/MDS panel), normal monocyte-committed CD64^+^ HPCs (tube 2 of the EF AML/MDS panel), and normal erythroid-committed CD36^+^ CD71^+^ CD33^−^ HPCs (tube 3 of the EF AML/MDS panel). For tube 1 of the EF AML/MDS panel, the first principal component (PC1, *x*-axis) showed the major contributions of the SSC (19%) and FSC (17%) parameters, whereas the second principal component (PC2, *y*-axis) showed the major contributions of HLA-DR (44%), CD34 (19%), and CD13 (10%) for the separation of the *t*(15;17) AML group from normal neutrophil-committed HPCs and all other AML groups.

For tube 2 of the EF AML/MDS panel, the most discriminating markers identified in PC1 were CD64 (18%), FSC (14%), and SSC (12%), whereas PC2 included HLA-DR (43%) and CD34 (21%), resulting in the clear distinction between *t*(15;17) AML blasts, normal monocyte-committed HPCs, and blasts from other AML categories. For tube 3 of the EF AML/MDS panel, PC1 indicated that the most useful parameters for group separation were FSC (13%) and SSC (11%), whereas PC2 showed that the best discriminating factors for tube 3 of the EF AML/MDS panel were HLA-DR (32%), CD34 (21%), and CD33 (13%), allowing for good separation between *t*(15;17) AML blasts, normal erythroid-committed HPCs, and blasts from other AML categories.

### 3.4 Compass Database-Guided DfN-MRD Evaluation in AML With Recurrent Genetic Abnormalities

In our study, the aberrant expression of lymphocytic markers on AML blasts was observed in a small number of cases: CD56 expression was observed in 13% of *t*(15;17) AML cases; the coexpression of CD19 and CD56 was observed in 28% of *t*(8;21) AML cases; the partial and low expression of CD19 was observed in 42% of *t*(8;21) AML cases; CD4 expression was observed in 7% of inv(16)/*t*(16;16) AML cases and in 18% of *MLL-r* AML cases; and CD7 expression was observed in 9% of all AML cases. Thus, performing MRD follow-up using these phenotypic aberrancies would only be applicable to a limited number of AML cases.

Therefore, we evaluated the utility of applying the Compass database-guided analysis based on the first three tubes of the EF AML/MDS panel to the detection of residual blasts in four AML cases harboring the most frequently detected genetic abnormalities.

#### 3.4.1 Compass Database-Guided Analysis for the Detection of APL Residual Blasts by MFC Versus qRT-PCR Evaluation

Although the qRT-PCR evaluation of the *PML-RARA* fusion transcript of 10^−4^ was more sensitive, and a difference of 1 log_10_ was noticed between the qRT-PCR and those obtained for the MRD evaluation by MFC (**Figure S2**), the clinical relevance of these results was the same according to ELN MRD Working Party consensus, which recommended a threshold of 0.1% to distinguish “MRD-positive” from “MRD-negative” patients by MFC (4).

#### 3.4.2 Compass Database-Guided Analysis for the Detection of *MLL*-r Residual Blasts by MFC Versus qRT-PCR Evaluation

For the *MLL*-r AML case, higher levels of residual blasts were detected by MFC than by *WT1* qRT-PCR at two time points before cytologic relapse occurred, and in other three time points, the values were aligned (**Figure S3**).

#### 3.4.3 Compass Database-Guided Analysis for the Detection of *t*(8;21) Residual Blasts by MFC Versus qRT-PCR Evaluation

A better prediction for disease progression was achieved using the MFC-MRD evaluation compared with the qRT-PCR *RUNX1-RUNX1T1* evaluation in the *t*(8;21) AML case (**Figure S4**).

#### 3.4.4 Compass Database-Guided Analysis for the Detection of inv(16) Residual Blasts by MFC Versus qRT-PCR Evaluation

The interpretation of DfN MFC data was most difficult for the inv(16) AML case because the isolation of events corresponding to residual blasts required the evaluation of multiple clusters on t-distributed stochastic neighbor embedding (t-SNE) graphs. In addition, in this case, we noticed discordances between the results obtained using the different EF AML/MDS tubes at the same time points (**Figure S5**). Despite these complications, overall, a good correlation was observed between the results obtained from the qRT-PCR and detection by MFC for identifying *CBFB-MYH11* and MRD.

## 4 Discussion

The immunophenotypic characterization of AML blasts by MFC, combined with the morphological examination of BM aspirates, plays a critical role in the initial AML diagnosis and classification into different French–American–British (FAB) subgroups, which have been used for AML classification since 1976 (15).

However, relatively little is known regarding the associations between genetic alterations and distinct immunophenotypic profiles.

A lack of consensus regarding the choosing of AML immunophenotyping panels and data analysis strategies represent major drawbacks for AML classification. A collaborative effort within the EF consortium led to the development of the EF AML/MDS panel, which allows for the unequivocal identification of AML blasts and lineage assignments and the accurate evaluation of myeloid lineage maturation profiles (5, 9, 10). However, the discriminatory potential for this panel to differentiate between AML blasts and normal my-HPCs, in addition to the utility of the panel for AML blast classification across different cytogenetic groups, remains poorly explored.

Novel software for MFC data analysis has been developed by the EF group, which includes several analysis tools to facilitate phenotypic comparisons between pathological cells derived from different groups of diseases, with the ultimate aim of facilitating the performance of fast, objective, and reproducible diagnostic assessments. A database-guided analysis of MFC outcomes is capable of objectively analyzing all single leukemic events and classifying each event individually, providing a global image of the composition of the leukemic bulk, which can frequently be heterogeneous in AML (16). This method performs accurate, simultaneous measurements of the mean fluorescence values from eight different markers, and two scatter parameters (FSC and SSC) represent a more objective approach than expert-based interpretations, which considers arbitrary categorical classifications of negative versus positive and low versus bright patterns of marker expression for the dominant leukemic population (17).

The Compass database-guided analysis, which is based on a PCA algorithm, has been demonstrated to be effective in various studies, such as for acute leukemia orientation (16), B‐cell chronic lymphoproliferative disorder classification (18), multiple myeloma diagnosis and monitoring (19), B-cell acute lymphoblastic leukemia follow-up (20), and MRD assessment in older patients with AML (21).

In this study, we sought to evaluate whether this method and the antibody combinations provided by the first three tubes of the EF AML/MDS panel could be used to identify the most frequently identified recurrent genetic abnormalities in AML to rapidly orient cytogenetic and molecular tests and highlight the DfN characteristics of AML blast immunophenotypes.

Using the algorithm described here, the Compass database-guided diagnosis enabled the correct classification of AML types with an AUC of 0.83 (95% CI: 0.75–0.89; *p* < 0.001). The major advantage of this method was the ability to exclude certain recurrent genetic abnormalities from new AML cases, with 92% sensitivity and a 98.5% negative predictive value, which can prevent the performance of unnecessary, expensive, and time-consuming tests.

This method also allowed for the identification of *NPM1*-mutant AML cases with an APL-like immunophenotype, which might benefit from personalized therapy.

In addition, using this method, we observed a significant phenotypic heterogeneity among the various *MLL*-r AML cases, which is consistent with the prognostic variability associated with this AML type. This method could be useful for identifying immunophenotypic patterns associated with different translocations involving the *MLL* gene.

The application of the PCA-based algorithm to the four typical AML groups with recurrent genetic abnormalities that were included in the reference database highlighted the relative contributions of each marker included in the staining panel. The increased contributions of the SSC and FSC parameters highlighted the importance of rigorously standardizing these parameters over time and across instruments when performing multicenter studies.

In addition to SSC and FSC, the CD34, HLA-DR, CD13, CD64, and CD33 markers were identified as useful for separation among the four AML disease categories and for the DfN identification. Therefore, combining these markers into a single tube could be useful for improving both the identification of AML groups with recurrent genetic abnormalities and DfN monitoring.

However, a recently published study showed that the antibodies included in the first five tubes of the EF AML/MDS panel were not sufficiently effective to discriminate between normal my-HPCs and AML blasts. Phenotypically, normal CD34^+^ HPCs isolated by fluorescence-activated cell sorting from patients with undetectable MRD possess substantial genetic abnormalities. Therefore, the identification of more specific leukemic antigens, together with improvements in MRD sensitivity using MFC and NGS data, remains necessary for the implementation of individualized treatments to prolong survival among older patients with AML (21).

Our preliminary data show that the Compass database-guided analysis represents a helpful tool for the identification of DfN, which may be useful for advancing MRD evaluations in AML.

According to our data, a similar approach has been applied for the detection of DfN patterns during MRD assessments in older AML patients. This method allowed the detection of DfN patterns and complete remission by MFC-MRD before morphological complete remission, serving as an independent prognostic factor in older AML patients. Therefore, this method can improve the sensitivity of MRD detection after semi-intensive therapy or hypomethylating agents (21).

The identification of phenotypic imprints for AML groups with recurrent genetic abnormalities may allow for the detection of leukemic blasts, even in the absence of phenotypic identification at diagnosis.

The antibody combination from the first three tubes of the EF AML/MDS panel has differential efficiencies for assessing MRD across different types of AML. Therefore, the use of multiple tubes could increase the efficiency of MRD evaluation using MFC.

The detection of AML MRD using the Compass database-guided analysis was superior to *WT1* qRT-PCR evaluation for predicting the risk of disease relapse in *MLL*-r AML.

In addition, previously published data show that qRT-PCR tests failed to predict disease progression in *t*(8;21) AML cases with low levels of MRD (22). In line with this study (22), we observed that the MFC method showed a constant low level of MRD positivity before relapse in the *t*(8;21) AML case, unlike the qRT-PCR method that indicated MRD levels below 10^−4^ at three different time points.

A good correlation was also observed between MRD evaluation by qRT-PCR and MFC in *t*(15;17) AML and inv(16) AML cases.

These preliminary results show that MRD evaluation in AML using MFC combined with Compass database-guided analysis should be considered to complement molecular biology for MRD detection.

The limited number of antibodies that can be combined into a single tube for routine MFC diagnostic purposes is a disadvantage of this method, as a relatively high number of unclassified events remains in the CD45^+low^ gate, limiting the specificity and sensitivity of this method.

However, the findings of this study require large-scale validation in a multicenter study to gather sufficient quantities of sample data to establish a robust analytical model that can contribute to the development of supervised machine learning techniques capable of performing automated MFC interpretation for the objective detection of MRD in AML.

In conclusion, the first three tubes of the EF AML/MDS panel, combined with the Compass database-guided analysis:

- allows for the exclusion of frequent recurrent genetic abnormalities in new AML cases, with 92% sensitivity and a 98.5% negative predictive value, which can contribute to preventing the performance of unnecessary, expensive, and time-consuming tests;
- allows for the correct classification of *t*(15;17), *t*(8;21), inv(16)/*t*(16;16), and *MLL*-r AML groups with an AUC of 0.83 (95% CI: 0.75–0.89; *p* < 0.00);
- provides valuable clues and can direct the extensive genetic or molecular exploration of difficult cases, such as *MLL*-r AML;
- allows for the identification of *NPM1* ^+^ AML cases that have an APL-like phenotype and may benefit from ATRA therapy; and
- allows for the identification of DfN patterns, which may be useful for advancing MRD evaluations in AML.

## Data Availability Statement

The original contributions presented in the study are publicly available. This data can be found here: FlowRepository, ID: FR-FCM-Z3JL.

## Ethics Statement

Written informed consent was obtained from each patient and healthy donor (HD), as approved by the institutional procedures of the independent ethics committee and the Comité de Protection des Personnes - Ile de France (NCT03233074/17.07.2017). The patients/participants provided their written informed consent to participate in this study.

## Author Contributions

Conceptualization: C-MA. Formal analysis: C-MA, RV-M, CS, MM, AT, LR, PF-G, AS, and MC. Validation: C-MA, RV-M, CS, MM, AT, LR, PF-G, AS, and MC. Clinical investigation: ET, DG, ad CT. Data curation: C-MA. Writing—original draft preparation: C-MA. Writing—review and editing: C-MA, RV-M, CS, MM, AT, LR, PF-G, AS, MC, ET, CT, LC, and DG. All authors have read and agreed to the published version of the manuscript.

## Funding

Funding was provided by the Association Les Amis de Rémi (France).

## Conflict of Interest

The authors declare that the research was conducted in the absence of any commercial or financial relationships that could be construed as a potential conflict of interest.

## Publisher’s Note

All claims expressed in this article are solely those of the authors and do not necessarily represent those of their affiliated organizations, or those of the publisher, the editors and the reviewers. Any product that may be evaluated in this article, or claim that may be made by its manufacturer, is not guaranteed or endorsed by the publisher.
