# Supplementary material for: Database-Guided Analysis for Immunophenotypic Diagnosis and Follow-Up of Acute Myeloid Leukemia With Recurrent Genetic Abnormalities
Source: Front Oncol. 2021 Nov 5;11:746951. doi: 10.3389/fonc.2021.746951 (PMC8602100; doi:10.3389/fonc.2021.746951)
Supplement: Supplementary file 1 [file Presentation_1.pptx]

## Slide 1
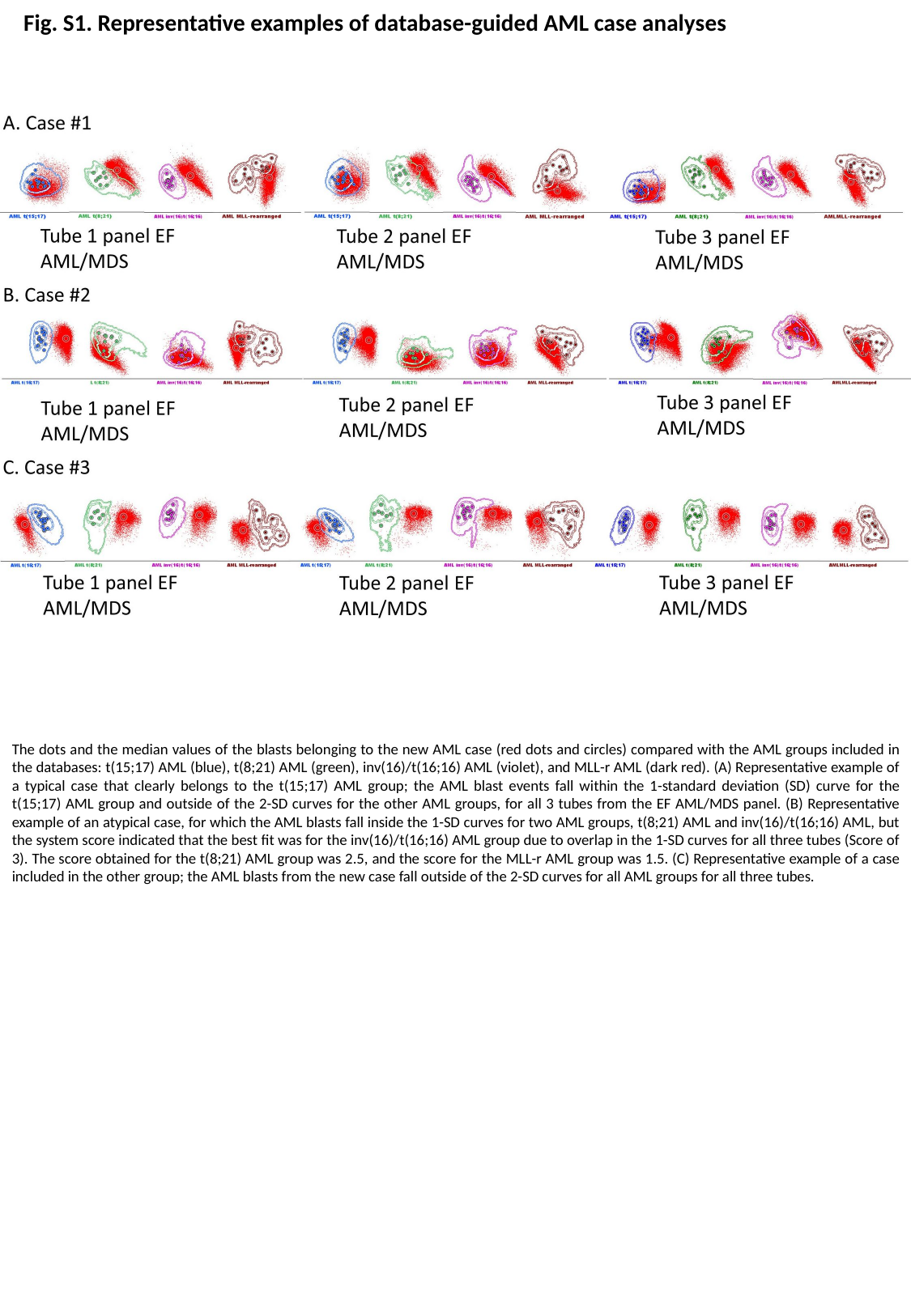

Fig. S1. Representative examples of database-guided AML case analyses
The dots and the median values of the blasts belonging to the new AML case (red dots and circles) compared with the AML groups included in the databases: t(15;17) AML (blue), t(8;21) AML (green), inv(16)/t(16;16) AML (violet), and MLL-r AML (dark red). (A) Representative example of a typical case that clearly belongs to the t(15;17) AML group; the AML blast events fall within the 1-standard deviation (SD) curve for the t(15;17) AML group and outside of the 2-SD curves for the other AML groups, for all 3 tubes from the EF AML/MDS panel. (B) Representative example of an atypical case, for which the AML blasts fall inside the 1-SD curves for two AML groups, t(8;21) AML and inv(16)/t(16;16) AML, but the system score indicated that the best fit was for the inv(16)/t(16;16) AML group due to overlap in the 1-SD curves for all three tubes (Score of 3). The score obtained for the t(8;21) AML group was 2.5, and the score for the MLL-r AML group was 1.5. (C) Representative example of a case included in the other group; the AML blasts from the new case fall outside of the 2-SD curves for all AML groups for all three tubes.

## Slide 2
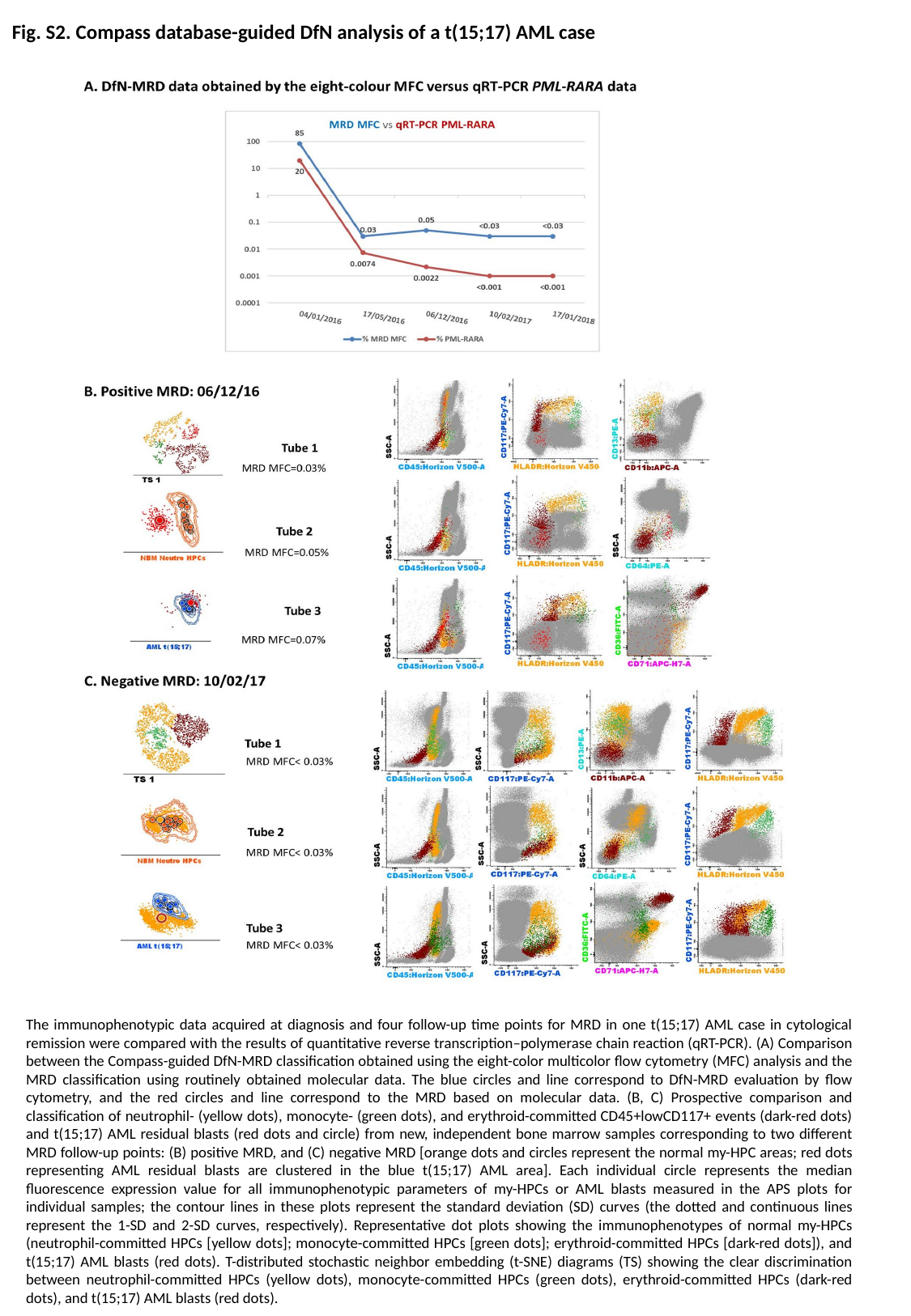

Fig. S2. Compass database-guided DfN analysis of a t(15;17) AML case
The immunophenotypic data acquired at diagnosis and four follow-up time points for MRD in one t(15;17) AML case in cytological remission were compared with the results of quantitative reverse transcription–polymerase chain reaction (qRT-PCR). (A) Comparison between the Compass-guided DfN-MRD classification obtained using the eight-color multicolor flow cytometry (MFC) analysis and the MRD classification using routinely obtained molecular data. The blue circles and line correspond to DfN-MRD evaluation by flow cytometry, and the red circles and line correspond to the MRD based on molecular data. (B, C) Prospective comparison and classification of neutrophil- (yellow dots), monocyte- (green dots), and erythroid-committed CD45+lowCD117+ events (dark-red dots) and t(15;17) AML residual blasts (red dots and circle) from new, independent bone marrow samples corresponding to two different MRD follow-up points: (B) positive MRD, and (C) negative MRD [orange dots and circles represent the normal my-HPC areas; red dots representing AML residual blasts are clustered in the blue t(15;17) AML area]. Each individual circle represents the median fluorescence expression value for all immunophenotypic parameters of my-HPCs or AML blasts measured in the APS plots for individual samples; the contour lines in these plots represent the standard deviation (SD) curves (the dotted and continuous lines represent the 1-SD and 2-SD curves, respectively). Representative dot plots showing the immunophenotypes of normal my-HPCs (neutrophil-committed HPCs [yellow dots]; monocyte-committed HPCs [green dots]; erythroid-committed HPCs [dark-red dots]), and t(15;17) AML blasts (red dots). T-distributed stochastic neighbor embedding (t-SNE) diagrams (TS) showing the clear discrimination between neutrophil-committed HPCs (yellow dots), monocyte-committed HPCs (green dots), erythroid-committed HPCs (dark-red dots), and t(15;17) AML blasts (red dots).

## Slide 3
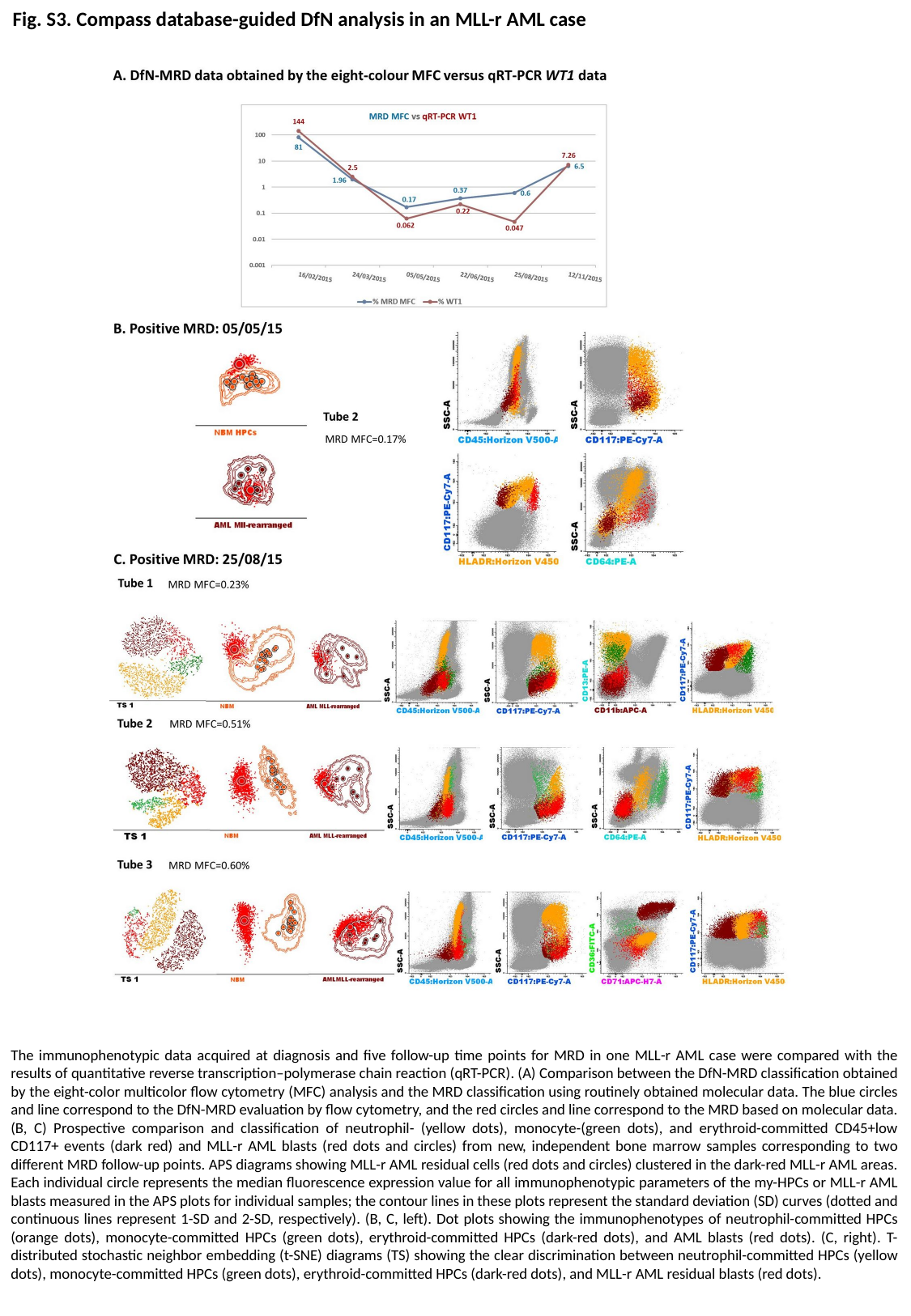

Fig. S3. Compass database-guided DfN analysis in an MLL-r AML case
The immunophenotypic data acquired at diagnosis and five follow-up time points for MRD in one MLL-r AML case were compared with the results of quantitative reverse transcription–polymerase chain reaction (qRT-PCR). (A) Comparison between the DfN-MRD classification obtained by the eight-color multicolor flow cytometry (MFC) analysis and the MRD classification using routinely obtained molecular data. The blue circles and line correspond to the DfN-MRD evaluation by flow cytometry, and the red circles and line correspond to the MRD based on molecular data. (B, C) Prospective comparison and classification of neutrophil- (yellow dots), monocyte-(green dots), and erythroid-committed CD45+low CD117+ events (dark red) and MLL-r AML blasts (red dots and circles) from new, independent bone marrow samples corresponding to two different MRD follow-up points. APS diagrams showing MLL-r AML residual cells (red dots and circles) clustered in the dark-red MLL-r AML areas. Each individual circle represents the median fluorescence expression value for all immunophenotypic parameters of the my-HPCs or MLL-r AML blasts measured in the APS plots for individual samples; the contour lines in these plots represent the standard deviation (SD) curves (dotted and continuous lines represent 1-SD and 2-SD, respectively). (B, C, left). Dot plots showing the immunophenotypes of neutrophil-committed HPCs (orange dots), monocyte-committed HPCs (green dots), erythroid-committed HPCs (dark-red dots), and AML blasts (red dots). (C, right). T-distributed stochastic neighbor embedding (t-SNE) diagrams (TS) showing the clear discrimination between neutrophil-committed HPCs (yellow dots), monocyte-committed HPCs (green dots), erythroid-committed HPCs (dark-red dots), and MLL-r AML residual blasts (red dots).

## Slide 4
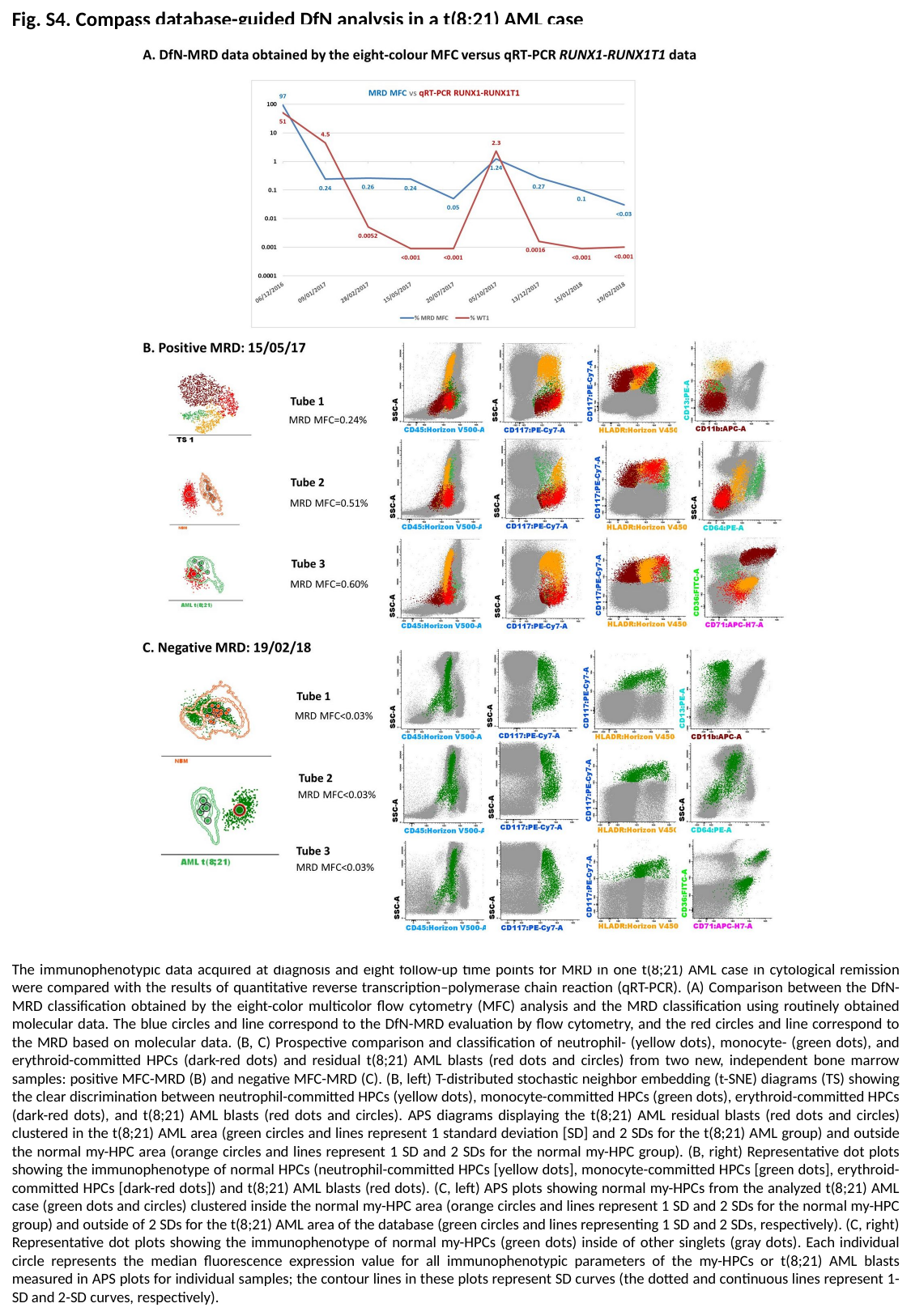

Fig. S4. Compass database-guided DfN analysis in a t(8;21) AML case
The immunophenotypic data acquired at diagnosis and eight follow-up time points for MRD in one t(8;21) AML case in cytological remission were compared with the results of quantitative reverse transcription–polymerase chain reaction (qRT-PCR). (A) Comparison between the DfN-MRD classification obtained by the eight-color multicolor flow cytometry (MFC) analysis and the MRD classification using routinely obtained molecular data. The blue circles and line correspond to the DfN-MRD evaluation by flow cytometry, and the red circles and line correspond to the MRD based on molecular data. (B, C) Prospective comparison and classification of neutrophil- (yellow dots), monocyte- (green dots), and erythroid-committed HPCs (dark-red dots) and residual t(8;21) AML blasts (red dots and circles) from two new, independent bone marrow samples: positive MFC-MRD (B) and negative MFC-MRD (C). (B, left) T-distributed stochastic neighbor embedding (t-SNE) diagrams (TS) showing the clear discrimination between neutrophil-committed HPCs (yellow dots), monocyte-committed HPCs (green dots), erythroid-committed HPCs (dark-red dots), and t(8;21) AML blasts (red dots and circles). APS diagrams displaying the t(8;21) AML residual blasts (red dots and circles) clustered in the t(8;21) AML area (green circles and lines represent 1 standard deviation [SD] and 2 SDs for the t(8;21) AML group) and outside the normal my-HPC area (orange circles and lines represent 1 SD and 2 SDs for the normal my-HPC group). (B, right) Representative dot plots showing the immunophenotype of normal HPCs (neutrophil-committed HPCs [yellow dots], monocyte-committed HPCs [green dots], erythroid-committed HPCs [dark-red dots]) and t(8;21) AML blasts (red dots). (C, left) APS plots showing normal my-HPCs from the analyzed t(8;21) AML case (green dots and circles) clustered inside the normal my-HPC area (orange circles and lines represent 1 SD and 2 SDs for the normal my-HPC group) and outside of 2 SDs for the t(8;21) AML area of the database (green circles and lines representing 1 SD and 2 SDs, respectively). (C, right) Representative dot plots showing the immunophenotype of normal my-HPCs (green dots) inside of other singlets (gray dots). Each individual circle represents the median fluorescence expression value for all immunophenotypic parameters of the my-HPCs or t(8;21) AML blasts measured in APS plots for individual samples; the contour lines in these plots represent SD curves (the dotted and continuous lines represent 1-SD and 2-SD curves, respectively).

## Slide 5
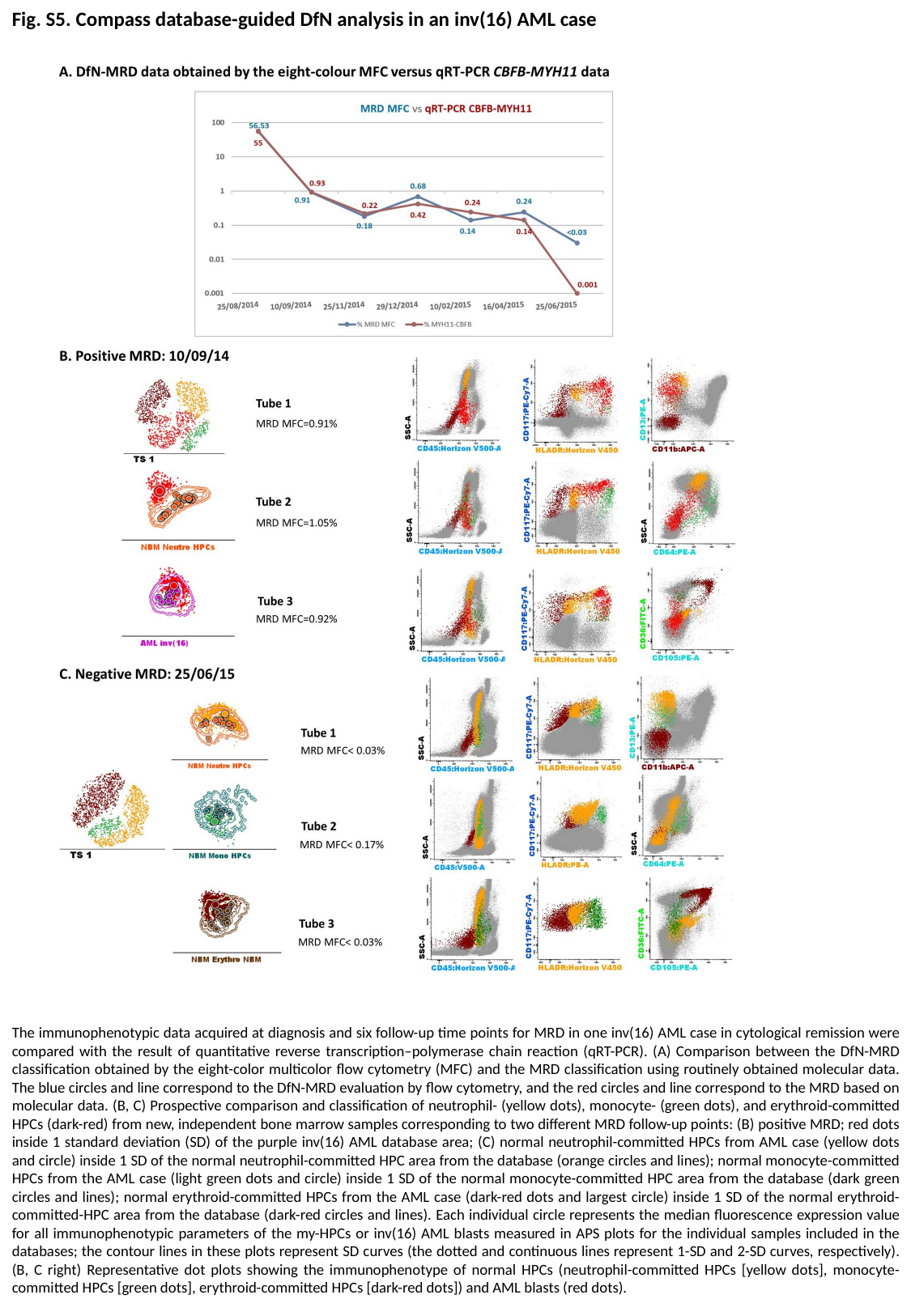

Fig. S5. Compass database-guided DfN analysis in an inv(16) AML case
The immunophenotypic data acquired at diagnosis and six follow-up time points for MRD in one inv(16) AML case in cytological remission were compared with the result of quantitative reverse transcription–polymerase chain reaction (qRT-PCR). (A) Comparison between the DfN-MRD classification obtained by the eight-color multicolor flow cytometry (MFC) and the MRD classification using routinely obtained molecular data. The blue circles and line correspond to the DfN-MRD evaluation by flow cytometry, and the red circles and line correspond to the MRD based on molecular data. (B, C) Prospective comparison and classification of neutrophil- (yellow dots), monocyte- (green dots), and erythroid-committed HPCs (dark-red) from new, independent bone marrow samples corresponding to two different MRD follow-up points: (B) positive MRD; red dots inside 1 standard deviation (SD) of the purple inv(16) AML database area; (C) normal neutrophil-committed HPCs from AML case (yellow dots and circle) inside 1 SD of the normal neutrophil-committed HPC area from the database (orange circles and lines); normal monocyte-committed HPCs from the AML case (light green dots and circle) inside 1 SD of the normal monocyte-committed HPC area from the database (dark green circles and lines); normal erythroid-committed HPCs from the AML case (dark-red dots and largest circle) inside 1 SD of the normal erythroid-committed-HPC area from the database (dark-red circles and lines). Each individual circle represents the median fluorescence expression value for all immunophenotypic parameters of the my-HPCs or inv(16) AML blasts measured in APS plots for the individual samples included in the databases; the contour lines in these plots represent SD curves (the dotted and continuous lines represent 1-SD and 2-SD curves, respectively). (B, C right) Representative dot plots showing the immunophenotype of normal HPCs (neutrophil-committed HPCs [yellow dots], monocyte-committed HPCs [green dots], erythroid-committed HPCs [dark-red dots]) and AML blasts (red dots).

## Slide 6
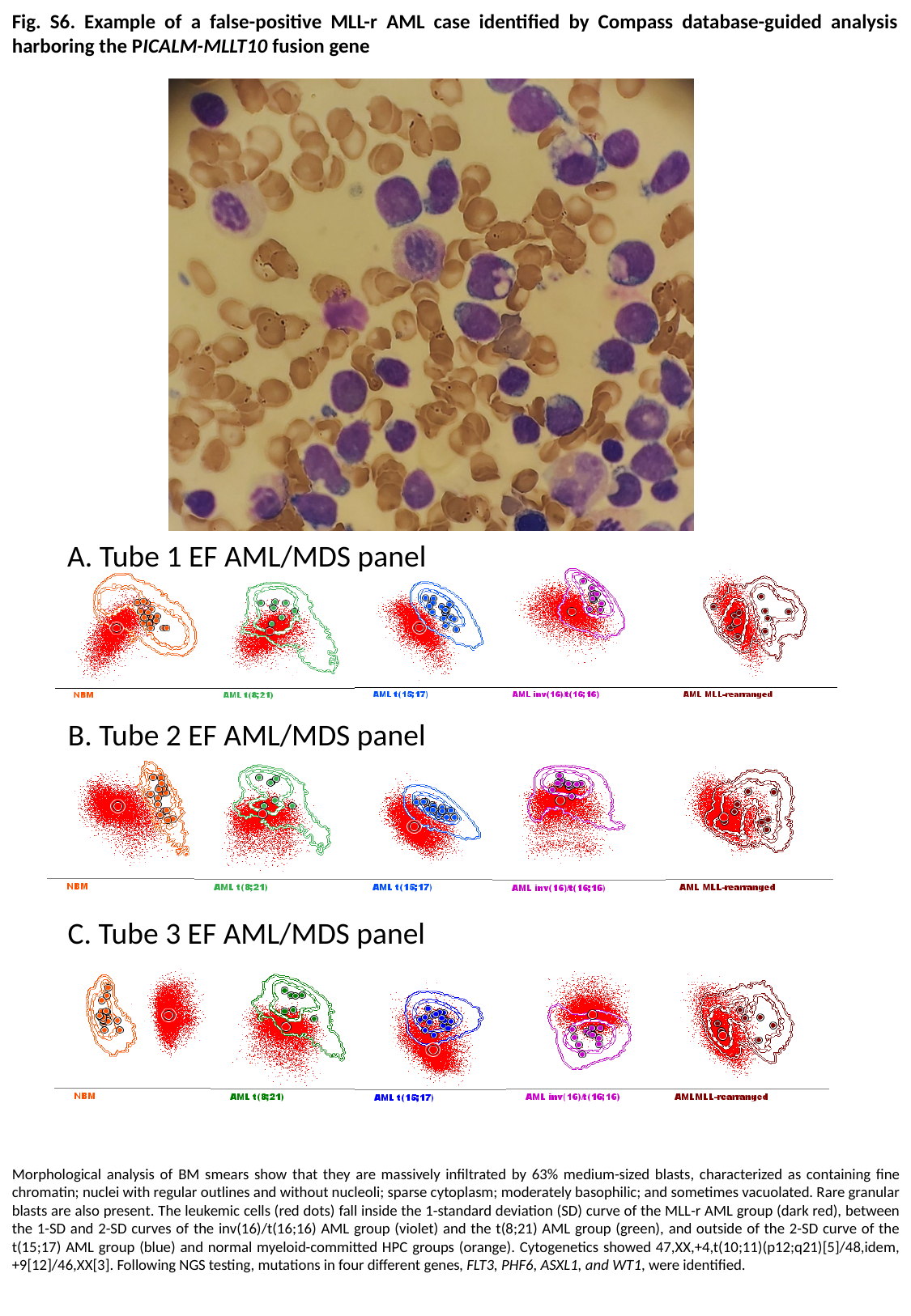

Fig. S6. Example of a false-positive MLL-r AML case identified by Compass database-guided analysis harboring the PICALM-MLLT10 fusion gene
A. Tube 1 EF AML/MDS panel
B. Tube 2 EF AML/MDS panel
C. Tube 3 EF AML/MDS panel
Morphological analysis of BM smears show that they are massively infiltrated by 63% medium-sized blasts, characterized as containing fine chromatin; nuclei with regular outlines and without nucleoli; sparse cytoplasm; moderately basophilic; and sometimes vacuolated. Rare granular blasts are also present. The leukemic cells (red dots) fall inside the 1-standard deviation (SD) curve of the MLL-r AML group (dark red), between the 1-SD and 2-SD curves of the inv(16)/t(16;16) AML group (violet) and the t(8;21) AML group (green), and outside of the 2-SD curve of the t(15;17) AML group (blue) and normal myeloid-committed HPC groups (orange). Cytogenetics showed 47,XX,+4,t(10;11)(p12;q21)[5]/48,idem,+9[12]/46,XX[3]. Following NGS testing, mutations in four different genes, FLT3, PHF6, ASXL1, and WT1, were identified.

## Slide 7
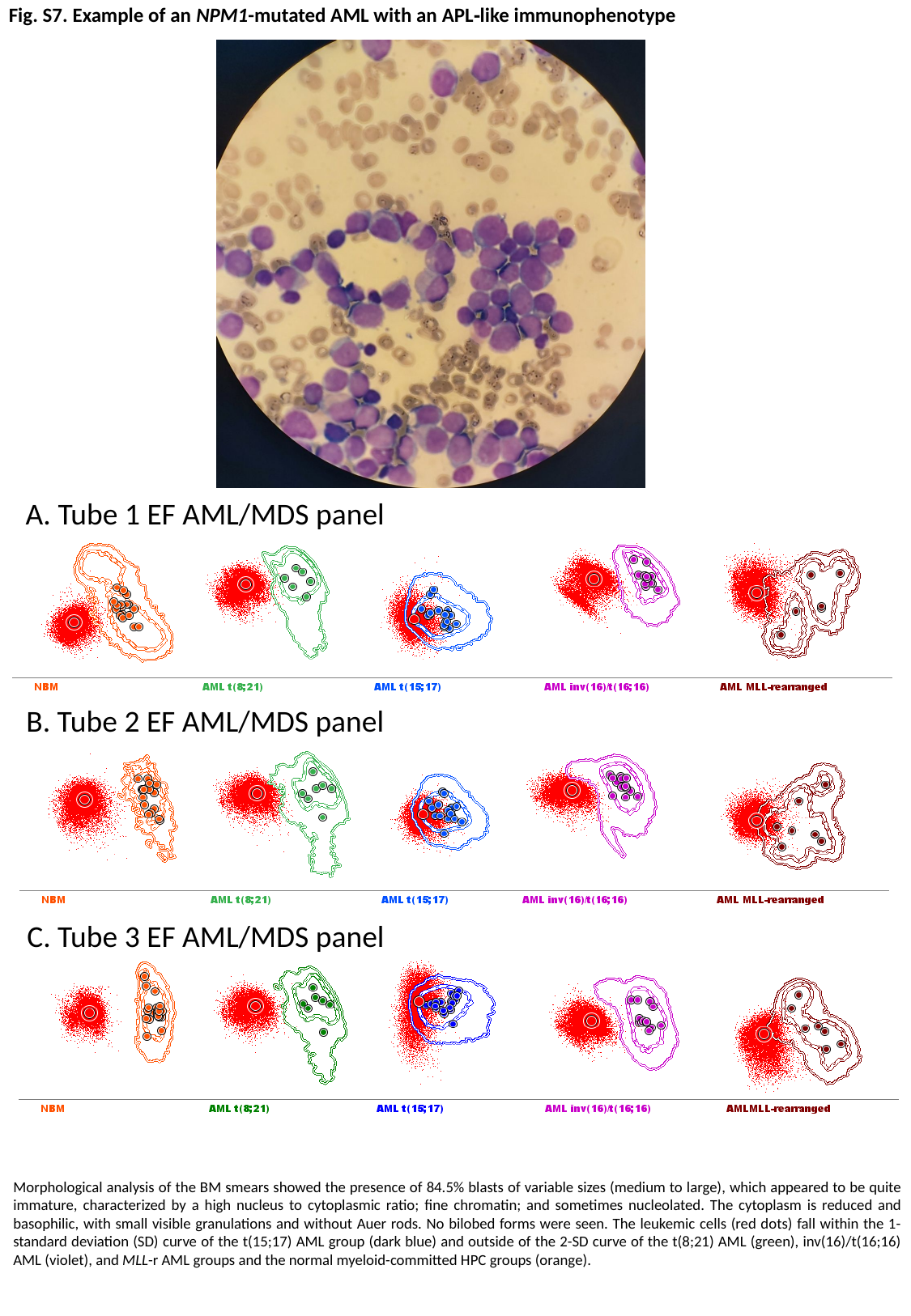

Fig. S7. Example of an NPM1-mutated AML with an APL‐like immunophenotype
A. Tube 1 EF AML/MDS panel
B. Tube 2 EF AML/MDS panel
C. Tube 3 EF AML/MDS panel
Morphological analysis of the BM smears showed the presence of 84.5% blasts of variable sizes (medium to large), which appeared to be quite immature, characterized by a high nucleus to cytoplasmic ratio; fine chromatin; and sometimes nucleolated. The cytoplasm is reduced and basophilic, with small visible granulations and without Auer rods. No bilobed forms were seen. The leukemic cells (red dots) fall within the 1-standard deviation (SD) curve of the t(15;17) AML group (dark blue) and outside of the 2-SD curve of the t(8;21) AML (green), inv(16)/t(16;16) AML (violet), and MLL-r AML groups and the normal myeloid-committed HPC groups (orange).
