# Supplementary material for: Database-Guided Analysis for Immunophenotypic Diagnosis and Follow-Up of Acute Myeloid Leukemia With Recurrent Genetic Abnormalities
Source: Front Oncol. 2021 Nov 5;11:746951. doi: 10.3389/fonc.2021.746951 (PMC8602100; doi:10.3389/fonc.2021.746951)
Supplement: Supplementary file 2 [file Table_1.doc]

Table S1. Detailed patients’ characteristics

| **Case** | **Gender** | **Age (Y)** | **WBC (10^9^/l)** | **Morphologic Diagnosis** | **WHO**  **Diagnosis** | **Karyotype/ Molecular analysis results** |  |
| --- | --- | --- | --- | --- | --- | --- | --- |
| AML 1 | M | 81 | 2.74 | AML-MRC | AML-MRC | 46,XY,del(4)(q22-24q31),del(5)(q13q34),del(17)(p11)[13]/46,XY[7] |  |
| AML 2 | F | 79 | 1.16 | AML NOS | AML NOS | 46,XX[25] |  |
| AML 3 | M | 87 | 1.43 | AML NOS | AML NOS | 45,X,-Y[9]/90, idem2[11] |  |
| AML 4 | M | 79 | 15.88 | AML-MRC | AML-MRC | 46,XY,-7,+8[19]/46,XY[1] |  |
| AML 5 | M | 81 | N/A | AML-MRC | AML-MRC | 45,XY,-5,-7,-7,add(11)(q23),-13,add(16)(p11),add(17)(p13),-19,+4mar[6]/46,XY[20] |  |
| AML 6 | M | 10 | 16.8 | APL | APL with PML-RARA | 46,XY,t(15;17)(q24;q21)[17]/46,XY[3] |  |
| AML 7 | M | 86 | 29.81 | AML NOS | AML NOS | 45,X,-Y,?del(12)(q?)[20] |  |
| AML 8 | F | 53 | 55.8 | AML NOS | AML with inv(16)(p13.1q22) or t(16;16)(p13.1;q22);CBFB-MYH11 | 46,XX,inv(16)(p13q22)[20] |  |
| AML 9 | F | 77 | 3.5 | AML NOS | AML NOS | 46,XX[20] |  |
| AML 10 | F | 66 | 1.25 | AML M6/M7? | AML-MRC | 44~45,XX,-5,add(8)(p11),-13,add(16)(p?),-17,add(17)(p11),-18,?add(21)(p11),+2~3mar[cp20] |  |
| AML 11 | M | 65 | 0.86 | AML-MRC | AML-MRC | 44~45,X,-Y,-16,-17,-18,-19,+3~4mar[18]/46,XY[2]  RUNX1 non-mutated |  |
| AML 12 | F | 77 | 7.86 | AML NOS | AML NOS | 46,XX,add(22)(p11)[20] |  |
| AML 13 | M | 77 | 5.15 | AML-MRC | AML-MRC | 46,XY,t(1;3)(p36;q21),del(8)(p11)[20] |  |
| AML 14 | M | 81 | 5.27 | AML M5 | AML with mutated NPM1 | 49,XY,+8x3[6]/47,XY,+8,der(4)t(4;13)(p16;q12)[10]/48,XY,+8x2,der(5)t(5;13)(p15;q12)[1],+der(8)t(8;13)(q24;q12)[1],der(10)t(10;13)(q26;q12)[1],der(14)t(13;14)(q12;p11)[1][cp4]/46,XY[2] |  |
| AML 15 | M | 47 | 10.38 | AML NOS | AML NOS | 46,XY[20] |  |
| AML 16 | F | 78 | N/A | AML NOS | AML-MRC | 47,XX,+9[8] |  |
| AML 17 | M | 43 | 84.42 | sAML | sAML | 46,XY,inv(2)(q31q36),dic(5;17)(q11;p11),+8,?add(9)(p?),ins(9;22)(q34;q11q1?2),del(13)(q12q14)[15]/49~71,idem,+5,+6,+8,+10,+11,+del(13)(q12q14),+14,+21,+21[cp6]/XY[1] |  |
| AML 18 | M | 22 | 12.13 | APL | APL with PML-RARA | 46,XY,t(15;17)(q24;q21),del(16)(q12-13)[20] |  |
| AML 19 | F | 69 | 42.58 | AML M5b | AML with mutated NPM1 | 46,XX |  |
| AML 20 | F | 16 | 46.56 | AML M5b | KMT2A(MLL)-rearranged AML | 46,XX,t(11;17)(q23;q21)[20] |  |
| AML 21 | F | 82 | 61.91 | AML M5b | AML with mutated RUNX1 | 46,XX[20] |  |
| AML 22 | F | 87 | 23.61 | AML NOS | AML NOS | 46,XX,t(1;6;11)(q13;q13;p15),der(3)(pter→q23::q27→qter),der(11)((11pter→11q12::3q26::3q23→3q27::11q12→11qter[20] |  |
| AML 23 | M | 78 | 1.71 | AML-MRC | AML-MRC | 48,XY,+1,+6,add(9)(q31-33),+10,add(12)(q24),del(16)(q?),-17,add(18)(q21)[12]/46,XY[8] |  |
| AML 24 | F | 88 | 8.23 | AML-MRC | AML-MRC | 45,XX,del(5)(q12q33),-7[20] |  |
| AML 25 | M | 77 | 4.69 | AML-MRC | AML-MRC | 45,XY,del(5)(q2?q33),-10,add(15)(q2?5),-17,+1~4mar[cp16]/46,XY[4] |  |
| AML 26 | M | 44 | 2.1 | AML M0 | Aggressive NK leukemia/lymphoma (ANKL) | 46,XY[20] |  |
| AML 27 | M | 19 | 139.93 | AML-MRC | AML-MRC | 47,XY,+8[18]/46,XY[2] |  |
| AML 28 | M | 77 | 7.59 | AML-MRC | AML with mutated NPM1 | 46,XY[20].nuc ish(KMT2A,CBFB,MYH11,TP53,NF1)x2[200] |  |
| AML 29 | F | 84 | 15.07 | AML-MRC | AML-MRC | 46,XX,del(20)(q11q13)[20] |  |
| AML 30 | M | 76 | 2.75 | AML NOS | AML NOS | 47,XY,+11[12]/46,XY[8].nuc ish(KMTAX3)[65/100],(MYH11,CBFBx2)[200]. Présence de 3 signaux de KMT2A dans 65% des noyaux analysés (par trisomie 11).L'analyse NGS montre la présence d'une mutation IDH2 p.R172K (c.515G>A), avec une VAF à 35%. |  |
| AML 31 | F | 55 | 4.07 | APL | APL with PML-RARA | 46,XX,t(15;17)(q24;q21)[17]/49,idem,+8,+14,+21[3] |  |
| AML 32 | F | 71 | 12.48 | AML M0 | sAML | 46,XX,t(9;22)(q34;q11)[1]/46,XX,t(9;22)(q34;q11),–18,+mar[19] |  |
| AML 33 | M | 60 | 23.29 | AML M5 | KMT2A(MLL)-rearranged AML | 48,XY,ins(10;11)(p12;q23q14),+2mar[1]/47,sl,-Y[11]/46~48,sl,t(1;21)(q42;q22),der(12)t(12;14)(p13;q23),-14,+1~3mar[10] |  |
| AML 34 | F | 57 | 93.02 | AML NOS | AML NOS | 46,XX[20] |  |
| AML 35 | F | 54 | 1.88 | AML NOS | AML with biallelic mutations of CEBPA | 46,XX[20]  RUNX1 non-mutated |  |
| AML 36 | M | 68 | 1.74 | AML M5b | AML with mutated RUNX1 | 52,XY,+10,+11,+12,+13,+14,+21[9]/46,XY[11] |  |
| AML 37 | F | 13 | 6.53 | AML M0 | AML NOS | 47,XX,+4,t(10;11)(p12;q21)[5]/48,idem,+9[12]/46,XX[3] |  |
| AML 38 | M | 38 | 128.22 | APLv | APL with PML-RARA | 46,XY,t(15;17)(q24;q21)[20] |  |
| AML 39 | F | 82 | 9.6 | AML M4 | sAML | 46,XX[20] |  |
| AML 40 | M | 57 | 5.21 | APL | AML with mutated NPM1 | 46,XY[20] |  |
| AML 41 | F | 85 | 150.6 | AML NOS | AML NOS | 46,XX,?del(14)(q?)[20] |  |
| AML 42 | M | 73 | 3.72 | t-AML | KMT2A(MLL)-rearranged AML | 46,XY,t(11;19)(q23;p13),-11,+der(11)t(11;19)[20] |  |
| AML 43 | F | 17 | 7.74 | AML NOS | AML with t(8;21)(q22;q22.1);RUNX1-RUNX1T1 | 46,XX,t(8;21)(q22;q22)[6]/45,idem,-X[3]/45,idem,-X,del(9)(q12q21-22)[7]/46,XX[4].nuc ish (RUNX1,RUNX1T1)x3(RUNX1 con RUNXTx2)[84/100],(NUP98)x2[196/200],(KMT2A)x2[197/200],(CBFB,MYH11)x2[200] |  |
| AML 44 | M | 66 | 21.1 | AML M4/M5 | AML with mutated NPM1 | 46,XY[20].nuc ish(KMT2A)x2 [199/200], (CBFB, MYH11]x2[200] |  |
| AML 45 | M | 83 | 1.27 | AML M4 | AML-MRC | 46,XY,del(9)(q21q34) or add(9)(q21)[2]/45,idem,del(5)(q12q34),-21[14]/46,XY[4] |  |
| AML 46 | F | 58 | 1.15 | AML M0/M1/M7? | AML with mutated RUNX1 | 46,XX[20].nucish(MECOM,RUNX1,CBFB, MYH11,RUNX1T1)x2[200], (KMT2Ax2)[197/200] |  |
| AML 47 | F | 87 | 28.97 | AML-MRC | AML-MRC | 46,XX[20] |  |
| AML 48 | M | 76 | 94.27 | AML M5 | AML NOS | 47,XY,+i(11)(p10) or +mar[16]/46,XY[4] |  |
| AML 49 | F | 62 | 4.4 | AML M0 | AML NOS | 46,XX[20] |  |
| AML 50 | F | 80 | 138.43 | AML M0 | AML with mutated NPM1 | 46,XX,del(20)(q11q13)[20] |  |
| AML 51 | M | 63 | 1 | AML NOS | AML NOS | 46,XY[20] |  |
| AML 52 | M | 74 | 5.59 | AML-MRC | AML with mutated RUNX1 | 46,XY[20] |  |
| AML 53 | F | 54 | 0.66 | AML-MRC | AML with mutated RUNX1 | 46,XX[25] |  |
| AML 54 | M | 70 | 6.93 | AML M0 | Blastic plasmacytoid dendritic cell neoplasm (BPDCN) | 46,XY[25] |  |
| AML 55 | M | 86 | 2.38 | AML-MRC | AML-MRC | 45,XY-7[20] |  |
| AML 56 | F | 84 | 5.27 | AML-MRC | AML-MRC | 44,XX,der(2)?t(2;17)(p11;q11),-5,add(11)(p11),-17,-17,-18,+mar,+dmin[5]/42,XX,der(2)?t(2;17)(p11;q11),-5,der(11)t(11;14)(p1?;q1?),der(13)add(13)(p11)del(13)(q?),-14-17,-17,-18,+mar[4]/46,XX[11] |  |
| AML 57 | F | 64 | 2.19 | AML-MRC | AML-MRC | 46,XX[20].nuc ish(KMT2A,MYH11,CBFB)x2[200] |  |
| AML 58 | F | 87 | N/A | AML NOS | AML NOS | 46,XX[20] |  |
| AML 59 | M | 80 | 2.76 | AML-MRC | AML-MRC | 45,XY,-7[3],XY[20].nuc ish[D7Z1,D5S2926,EZH2)x1[65/200],(KMT2A,MYH11,CBFB)x2[200] |  |
| AML 60 | M | 63 | 5.68 | AML M4 Eo | AML with inv(16)(p13.1q22) or t(16;16)(p13.1;q22);CBFB-MYH11 | 46,XY,inv(16)(p13q22)[20].nuc ish[MYH11, CBFB]x3 [MYH11 con CBFBx2][95/100],(KMT2Ax2][200] |  |
| AML 61 | M | 76 | 12.77 | AML M4 | AML with mutated NPM1 | 47,XY,+8[20]/48,idem,+der(8)?ind(8;?)(q13;?)[2]/46,XY[2].nuc ish(KMT2A,MYH11,CBFB)x2[200] |  |
| AML 62 | F | 71 | 42.1 | AML NOS | AML with mutated NPM1 | 46,XX[20] |  |
| AML 63 | F | 40 | 2.67 | AML M2 | AML NOS | 47,XX,+10[inc5]/46,XX[4] ECHEC |  |
| AML 64 | F | 54 | 537.85 | AML NOS | sAML | 46,XX,t(9;22;12)(q34;q11;q13)[12]/46,idem,-7,+r[8] |  |
| AML 65 | M | 84 | 109.65 | AML-MRC | AML-MRC | 46,XY[25] |  |
| AML 66 | M | 84 | 39.25 | AML-MRC | AML with mutated RUNX1 | 46,XY[20] |  |
| AML 67 | M | 85 | 17.42 | AML NOS | AML NOS | 46,XY[20] |  |
| AML 68 | F | 82 | N/A | AML-MRC | AML-MRC | 46,XX,del(5)(q13q31)[13]/45,idem,der(13;14)(q10;q10)[7] |  |
| AML 69 | M | 39 | 144.9 | AML M0 | AML with mutated NPM1 | 46,XY[25] |  |
| AML 70 | M | 61 | 33.04 | AML NOS | AML NOS | 48~49,XY,ins(10;11)(p1?2;q23q14),+2~3mar[25] |  |
| AML 71 | F | 74 | 79.31 | AML M4 | AML with mutated NPM1 | 46,XX[20] |  |
| AML 72 | M | 73 | 4.91 | AML NOS | AML NOS | 46,XY[25] |  |
| AML 73 | M | 51 | 2.44 | AML M0 | AML NOS | 46,XY[17] |  |
| AML 74 | F | 67 | 5.49 | AML-MRC | AML-MRC | 46,XX[20]  RUNX1 non-mutated |  |
| AML 75 | M | 60 | 119.63 | AML-MRC | AML with mutated NPM1 | 46,XY[20] |  |
| AML 76 | F | 87 | 1.02 | AML-MRC | AML-MRC | 47,XX,+10[13]/46,XX,del(7)(q2?1q32)[7] |  |
| AML 77 | M | 76 | 2.91 | AML M0 | AML-MRC | 46,XY,del(5)(q22q33),-7,-13,der(20)?del(20)(q?),+mar1x2[8]/46,idem,-der(20),+mar2[3]/46,XY[2] |  |
| AML 78 | M | 58 | 3.25 | AML-MRC | AML-MRC | 46,XY,t(3;21)(q26;q22)[20] |  |
| AML 79 | M | 72 | 31.7 | AML NOS | AML-MRC | 47,XY,+8[16]/46,XY[4] |  |
| AML 80 | M | 77 | 0.75 | AML-MRC | AML-MRC | 46,XY[14]/92,idemx2[6] |  |
| AML 81 | F | 88 | 2.95 | AML-MRC | AML-MRC | 46,XX[20] |  |
| AML 82 | M | 59 | 123.8 | AML M5a | AML-MRC | 47,XY,+11[9]/46,XY[11] |  |
| AML 83 | M | 60 | 23 | AML-MRC | AML-MRC | 45,XY,-7,inv(11)(p14q21)[17]/90,idemx2[2]/46,XY[1] |  |
| AML 84 | F | 70 | 7.51 | AML NOS | AML with t(8;21)(q22;q22.1);RUNX1-RUNX1T1 | 45,X,-X,t(8;21)(q22;q22)[9]/45,idem,der(2)t(2;8)(q32;q13)[11] |  |
| AML 85 | F | 35 | 17.42 | AML NOS | AML-MRC | 48,XX,+5,+8[20] |  |
| AML 86 | M | 76 | 185.55 | AML NOS | AML with mutated NPM1 | 46,XY[20] |  |
| AML 87 | F | 62 | 8.66 | AML NOS | AML-MRC | 45,XX,-7[6]/90,idemx2[12]/46,XX[2] |  |
| AML 88 | M | 63 | 4.94 | APL | APL with PML-RARA | 46,XY,t(15;17)(q24;q21)[11]/47,sl,+8[5]/46,sl,der(11)t(11;12)(p15;q13),del(12)(q13)[2]/46,XY[2] |  |
| AML 89 | M | 94 | 1.42 | AML NOS | AML NOS | 46,XY[20] |  |
| AML 90 | F | 94 | 19.17 | AML M4 | AML-MRC | 46,XX,del(13)(q13q22)[19]/46,XX[1] |  |
| AML 91 | F | 53 | 1.7 | AML-MRC | AML with mutated NPM1 | 45,X,-X[9]/46,[11] |  |
| AML 92 | M | 63 | 71.42 | AML NOS | AML NOS | 47,XY,+8[10]/94,idemx2[3]/47,idem,der(18)t(3;18)(q24;q23)[7] |  |
| AML 93 | M | 45 | 6.42 | AML-MRC | AML-MRC | 46,XY[20] |  |
| AML 94 | F | 85 | 3.98 | AML-MRC | AML-MRC | 46,XX,-1,add(3)(q?24),-5,-8,-10,-12,+5mar[12]/46,XX[8] |  |
| AML 95 | M | 68 | 2.54 | AML M2 | AML NOS | 47,XY,+14[8]/46,XY[12] |  |
| AML 96 | F | 57 | 7.47 | AML-MRC | AML-MRC | 47,XX,der(1)(9pter→9p11::1p22→1qter),+der(1),der(4)(9qter→9q33::?20q::4p1?5→4qter),der(9)(?::9p11→9q33::?),der(20)(20pter→20q11::1p22→1pter)[20] |  |
| AML 97 | F | 77 | 107.31 | AML NOS | AML NOS | 46,XX[20] |  |
| AML 98 | F | 21 | 9.29 | AML M0 | KMT2A(MLL)-rearranged AML | 46,XX,t(9;11)(p21;q23)[20] |  |
| AML 99 | F | 86 | 1.14 | AML-MRC | AML-MRC | 46,XX,del(5)(q13q31)[2]/45,sl,der(13;20)(q10;p10)[4]/45~46,sl1-2,-3,-del(5)(q13q31),-6,-8,-10,-11,add(11)(q2?2),-19,-21,+6~7mar[cp4] |  |
| AML 100 | F | 92 | N/A | AML-MRC | AML-MRC | 46,XX[20] |  |
| AML 101 | F | 47 | N/A | AML M4 | AML with mutated NPM1 | 46,XX[20] |  |
| AML 102 | F | 65 | N/A | AML M4 | AML with mutated NPM1 | 46,XX[30].ish 11q23(MLLx2)[20],16q22(CBFBx2)[20],17q21(RARAx2)[10].nuc ish(MLLx2)[200], (CBFBx2)[200],(RARAx2)[200] |  |
| AML 103 | F | 73 | N/A | AML M5 | AML with mutated NPM1 | 46,XY[20].ish 11q23(MLLx2)[20],16q22(CBFBx2)[20].nuc ish(MLLx2)[200],(CBFBx2)[200] |  |
| AML 104 | F | 26 | N/A | AML M2 | KMT2A(MLL)-rearranged AML | 46,XX,t(9;11)(p21;q23)[12]/47,sl,+der(9)t(9;11)[8].ish der(9)t(9;11)(3'MLL+)[10],t(9;11)(3'MLL+;5'MLL+)[18],16q22(CBFBx2) [20].nuc ish(5'MLLx2,3'MLLx3)(5'MLL con 3'MLLx1)[45/100]/(MLLx2)(5'MLL sep 3'MLLx1)[43/100],(CBFBx2)[200] |  |
| AML 105 | M | 32 | 1.06 | APLv | APL with PML-RARA | 46,XY,t(15;17)(q24;q21)[20] |  |
| AML 106 | F | 64 | N/A | APLv | APL with PML-RARA | *PML-RARA+* (bcr3 transcript) |  |
| AML 107 | M | 65 | 0.7 | APL | APL with PML-RARA | *PML-RARA+* (bcr1 transcript) |  |
| AML 108 | M | 33 | 4.79 | APLv | APL with PML-RARA | *PML-RARA+* (bcr1 transcript) |  |
| AML  109 | M | 32 | 6.62 | AML M2 | AML with t(8;21)(q22;q22.1);RUNX1-RUNX1T1 | *RUNX1-RUNX1T1 (AML1-ETO)+* |  |
| AML  110 | M | 33 | N/A | AML M2 | AML with t(8;21)(q22;q22.1);RUNX1-RUNX1T1 | *RUNX1-RUNX1T1 (AML1-ETO)+* |  |
| AML 111 | F | 29 | 2.83 | APLv | APL with PML-RARA | *PML-RARA+* (bcr3 transcript) |  |
| AML 112 | F | 43 | 206.15 | APLv | APL with PML-RARA | *PML-RARA+* (bcr3 transcript) |  |
| AML 113 | F | 57 | 41.96 | AML NOS | AML with t(8;21)(q22;q22.1);RUNX1-RUNX1T1 | *RUNX1-RUNX1T1 (AML1-ETO)+* |  |
| AML 114 | F | 64 | N/A | AML M5 | MLL-r PTD | *KMT2A*-PTD+ |  |
| AML 115 | M | 64 | 5.73 | APLv | APL with PML-RARA | *PML-RARA+* (bcr3 transcript) |  |
| AML 116 | M | 14 | 282.59 | APL | APL with PML-RARA | *PML-RARA+* (bcr3 transcript) |  |
| AML 117 | F | 51 | 7.73 | AML M2 | AML with t(8;21)(q22;q22.1);RUNX1-RUNX1T1 | *RUNX1-RUNX1T1 (AML1-ETO)+* |  |
| AML 118 | M | 43 | 40.41 | AML M2 | AML with t(8;21)(q22;q22.1);RUNX1-RUNX1T1 | *RUNX1-RUNX1T1 (AML1-ETO)+* |  |
| AML 119 | M | 32 | 327.13 | AML M4 Eo | AML with inv(16)(p13.1q22) or t(16;16)(p13.1;q22);CBFB-MYH11 | *CBFB-MYH11*+ |  |
| AML 120 | M | 23 | 112.33 | AML M4 Eo | AML with inv(16)(p13.1q22) or t(16;16)(p13.1;q22);CBFB-MYH11 | *CBFB-MYH11*+ |  |
| AML 121 | F | 69 | N/A | AML M4 Eo | AML with inv(16)(p13.1q22) or t(16;16)(p13.1;q22);CBFB-MYH11 | *CBFB-MYH11*+ |  |
| AML 122 | M | 37 | N/A | APL | APL with PML-RARA | *PML-RARA+* (bcr3 transcript) |  |
| AML 123 | M | 71 | 175.85 | AML M5 | MLL-r PTD | *KMT2A*-PTD+ |  |
| AML 124 | M | 73 | 1.51 | APLv | APL with PML-RARA | *PML-RARA+* (bcr1 transcript) |  |
| AML 125 | M | 68 68 | N/A | AML M4 Eo | AML with inv(16)(p13.1q22) or t(16;16)(p13.1;q22);CBFB-MYH11 | *CBFB-MYH11*+ |  |

t-AML, Acute myeloid leukemia, therapy related

sAML, secondary Acute myeloid leukemia

AML-MRC, Acute myeloid leukemia with myelodysplasia-related changes

AML NOS, Acute Myeloid Leukemia Not Otherwise Specified

APL, acute promyelocytic leukemia; APLv, acute promyelocytic leukemia variant

AML M6, Acute erythroid leukemia

AML M7, Acute megakaryocytic leukemia

AML M0, Minimally differentiated acute myeloblastic leukemia

AML M1, Acute myeloid leukemia without maturation

AML M2, Acute myeloblastic leukemia with maturation

AML M4, Myelomonocytic acute myeloid leukemia; M4 Eo, Acute myelomonocytic leukemia with eosinophilia

AML M5, Acute monoblastic/monocytic leukemia

AML M5a, Acute monoblastic leukemia

AML M5b, Acute monocytic leukemia

MLL-r PTD, Partial tandem duplication (PTD) of the KMT2A (MLL)
